# Supplementary material for: Continuous-variable fault-tolerant quantum computation under general noise
Source: Nat Commun. 2026 Feb 26;17:1709. doi: 10.1038/s41467-026-69036-5 (PMC12946304; doi:10.1038/s41467-026-69036-5)
Supplement: Supplementary file 1 — Supplementary Information [file 41467_2026_69036_MOESM1_ESM.pdf]

SUPPLEMENTARY INFORMATION

Supplementary Information of "Continuous-Variable Fault-Tolerant Quantum Computation under General Noise" is organized as follows. Supplementary Note 1 summarizes preliminaries and physical setups of continuous-variable (CV) quantum computation. In Supplementary Note 1 A, we review the stabilizer subsystem decomposition developed in Ref. [1]. Explicit representations of some CV quantum operations in this decomposition are also given. Supplementary Note 1 B defines physical operations to implement quantum circuits with CV systems. In Supplementary Note 1 C, we define the energy-constrained diamond norm, which will be used to measure how close a noisy map implemented by a physical circuit is to an ideal map. We also list its properties that are used in our fault-tolerance proof. In Supplementary Note 1 D, we define a noise model on CV systems against which we prove a threshold theorem in the later section. Supplementary Note 2 is the main part of constructing a fault-tolerant gadget and proving a threshold theorem. We construct fault-tolerant (FT) Gottesman-Kitaev-Preskill (GKP) gadgets in Supplementary Note 2 A and prove their fault-tolerant conditions in Supplementary Note 2 B. We further impose an energy-constraint condition for an FT gadget for the later analysis and check its validity in Supplementary Note 2 C. Finally, in Supplementary Note 2 D, we prove our main result, a threshold theorem for a CV circuit, in full detail.

Supplementary Note 1. SETTING

A. GKP code and stabilizer subsystem decomposition

A single-mode harmonic oscillator can be characterized by a pair of noncommutative quadrature operators $\hat{q} = \frac{1}{\sqrt{2}}(\hat{a} + \hat{a}^\dagger)$ and $\hat{p} = \frac{-i}{\sqrt{2}}(\hat{a} - \hat{a}^\dagger)$, satisfying $[\hat{q}, \hat{p}] = i$ (with $\hbar = 1$), acting on a separable Hilbert space $\mathcal{H}$ [2]. We consider the square-lattice GKP code for encoding a qubit into a harmonic oscillator. The ideal square-lattice GKP-encoded state (the GKP codeword) $|\overline{\psi}\rangle$ is formally defined as an infinite superposition of position eigenstates, i.e., $|\overline{\psi}\rangle = \alpha |\overline{0}\rangle + \beta |\overline{1}\rangle$ with

$$|\overline{j}\rangle := \sum_{s\in\mathbb{Z}} |\sqrt{\pi}(2s + j)\rangle_q \tag{1}$$

for $j = 0, 1$, where $|x\rangle_q$ satisfies $\hat{q}|x\rangle_q = x|x\rangle_q$ for the operator $\hat{q}$.

The GKP code can be regarded as a stabilizer code. To explain this, let $\hat{V}(v_1, v_2)$ be defined as

$$\hat{V}(v_1, v_2) := \exp[-i(v_1\hat{p} - v_2\hat{q})], \tag{2}$$

which leads to

$$\hat{V}(u_1, u_2)\hat{V}(v_1, v_2) = e^{i(v_1u_2 - u_1v_2)/2}\hat{V}(u_1 + v_1, u_2 + v_2). \tag{3}$$

Then, one can check that the state given in Eq. (1) is stabilized by the two stabilizer generators $\hat{S}_1$ and $\hat{S}_2$ given by

$$\hat{S}_1 = \hat{V}(2c, 0), \tag{4}$$

$$\hat{S}_2 = \hat{V}(0, 2c), \tag{5}$$

where we write for simplicity

$$c := \sqrt{\pi}. \tag{6}$$

The ideal GKP codeword $|\overline{j}\rangle$ is stabilized by elements of the stabilizer obtained from these stabilizer generators [3].

Most logical unitary gates on the GKP code can be realized by Gaussian unitaries, which are generated by first- or second-order polynomials of $\hat{q}$ and $\hat{p}$. In fact, the logical Pauli-X and -Z operators $\overline{X}$ and $\overline{Z}$ for this GKP code are given by

$$\overline{X} := \hat{V}(c, 0), \tag{7}$$

$$\overline{Z} := \hat{V}(0, c). \tag{8}$$

The logical Hadamard gate $\overline{H}$ can be implemented by the Fourier gate $\hat{F}$, which is defined as

$$\hat{F} := \hat{R}(\pi/2), \tag{9}$$

where

$$\hat{R}(\theta) := \exp[i\theta\hat{n}], \tag{10}$$

$$\hat{n} := (\hat{q}^2 + \hat{p}^2 - 1)/2. \tag{11}$$

The logical CNOT gate $\overline{\text{CNOT}}$ on the system 2 controlled by 1 can be implemented by the SUM gate, defined as

$$\text{SUM} := \exp(-i\hat{q}_1\hat{p}_2), \tag{12}$$

where $\hat{q}_i$ and $\hat{p}_i$ denote the quadrature operators in i^{th} system [3]. Other logical gates can be implemented through gate teleportations as long as one can prepare their magic states [4]. More precisely, the logical phase gate $\overline{S}$ can be implemented through the gate teleportation with the state $|\overline{Y}\rangle := (|\overline{0}\rangle + i\,|\overline{1}\rangle)/\sqrt{2}$, and the logical T gate $\overline{T}$ can be with the state $|\frac{\pi}{8}\rangle := (|\overline{0}\rangle + e^{\pi i/4}\,|\overline{1}\rangle)/\sqrt{2}$. Note that the logical phase gate $\overline{S}$ can also be implemented by a Gaussian unitary $\exp(i\hat{q}^2/2)$, which will not be used in this paper to simplify the analysis. Measurements in the logical Pauli-X or Z bases can be implemented, respectively, by homodyne detection in $\hat{p}$ or $\hat{q}$ quadrature followed by classical post-processing. The positive-operator-valued measure (POVM) of the q-homodyne detection is given by $|x\rangle\langle x|_q\,dx$. The classical post-processing is performed by a binning modulo $\sqrt{\pi}$ (the so-called GKP binning), and if binned to an even (respectively, odd) multiple of $\sqrt{\pi}$, the outcome is regarded as logical 0 (respectively, 1). These are brief summaries of a set of operations for implementing universal quantum computation with the GKP code.

There is a suitable representation for analyzing the GKP state and the GKP code, which we call the Zak representation [5–7]. This is explicitly pointed out in Ref. [8] and later used in several papers [9–11], while it has already appeared in an early study [12]. With this particular representation of a Hilbert space, Ref. [8] developed the subsystem decomposition technique, which introduces a tensor-product structure between the GKP logical subsystem and the rest (called a gauge mode in Ref. [8]). More recently, an alternative for this picture has been developed in Ref. [1]. Unlike the subsystem decomposition in Ref. [8], the subsystem decomposition in Ref. [1] has symmetry in position and momentum and has a direct connection to the ideal GKP decoding. For this reason, we will use the formalism of Ref. [1]. For completeness and ease of reference, we now review some of the results therein. Although the results in Ref. [1] apply to any logical dimensions and any number of modes, here we restrict our attention to the single-mode, square-lattice GKP qubit.

The Zak basis for the square-lattice GKP qubit is defined as [1]

$$|z_1, z_2\rangle_Z := \frac{e^{iz_1z_2/2}}{\sqrt{c}} \sum_{s\in\mathbb{Z}} e^{2ciz_2s}\,|z_1 + 2cs\rangle_q \tag{13}$$

$$= \hat{V}(z_1, z_2)\,|0, 0\rangle_Z\,, \tag{14}$$

for $z_1 \in [-\frac{c}{2}, \frac{3c}{2})$ and $z_2 \in [-\frac{c}{2}, \frac{c}{2})$. The expression above defines a unitary transformation between the Zak and position representations. (Mathematically, the Hilbert space for the former representation is $L^2\big([-\frac{c}{2}, \frac{3c}{2}) \times [-\frac{c}{2}, \frac{c}{2})\big)$.) As discussed in Ref. [11], we can enlarge the domain of (z_1, z_2) to $\mathbb{R}\times\mathbb{R}$ with the following quasi-periodic condition:

$$|z_1 + 2c, z_2\rangle_Z = e^{-ciz_2}\,|z_1, z_2\rangle_Z\,, \tag{15}$$

$$|z_1, z_2 + c\rangle_Z = e^{ciz_1/2}\,|z_1, z_2\rangle_Z\,. \tag{16}$$

Let $(\bar{z}_1, \bar{z}_2) \in \mathbb{R}\times\mathbb{R}$. Let $\lfloor\bar{z}\rceil_a$ be rounding $\bar{z}$ to an integer multiple of a and $\{\bar{z}\}_a$ be its residual, i.e.,

$$\lfloor\bar{z}\rceil_a := a\left\lfloor\frac{\bar{z}}{a} + \frac{1}{2}\right\rfloor, \tag{17}$$

$$\{\bar{z}\}_a := \bar{z} - \lfloor\bar{z}\rceil_a, \tag{18}$$

where $\lfloor\,\cdot\,\rfloor$ is the floor function. Then from Eqs. (15) and (16), we should interpret $|\bar{z}_1, \bar{z}_2\rangle_Z$ for $(\bar{z}_1, \bar{z}_2) \in \mathbb{R}\times\mathbb{R}$ as

$$|\bar{z}_1, \bar{z}_2\rangle_Z = e^{\frac{i}{2}(\lfloor\bar{z}_1\rceil_{2c}\lfloor\bar{z}_2\rceil_c - \lfloor\bar{z}_1\rceil_{2c}\{\bar{z}_2\}_c + \{\bar{z}_1\}_{2c}\lfloor\bar{z}_2\rceil_c)}\,|\{\bar{z}_1\}_{2c}, \{\bar{z}_2\}_c\rangle_Z\,. \tag{19}$$

Note that $\exp[\frac{i}{2}\lfloor\bar{z}_1\rceil_{2c}\lfloor\bar{z}_2\rceil_c] = \exp[-\frac{i}{2}\lfloor\bar{z}_1\rceil_{2c}\lfloor\bar{z}_2\rceil_c]$. The Zak basis satisfies the following orthogonal property for $z_1, z_1' \in [-\frac{c}{2}, \frac{3c}{2})$ and $z_2, z_2' \in [-\frac{c}{2}, \frac{c}{2})$:

$$\langle z_1, z_2|z_1', z_2'\rangle_Z = \delta(z_1 - z_1')\,\delta(z_2 - z_2'), \tag{20}$$

where $\delta(x)$ denotes the Dirac delta function. The Zak basis also satisfies the following completeness relation:

$$\int_{-\frac{c}{2}}^{\frac{3c}{2}} dz_1 \int_{-\frac{c}{2}}^{\frac{c}{2}} dz_2 \, |z_1, z_2\rangle\langle z_1, z_2|_Z = \hat{I}, \tag{21}$$

where $\hat{I}$ denotes the identity operator on the Hilbert space.

The ideal GKP state given in Eq. (1) is now given in the Zak basis as $|\overline{0}\rangle \propto |0,0\rangle_Z$ and $|\overline{1}\rangle \propto |c,0\rangle_Z$. This defines a qubit in an oscillator. However, these are not states in the usual sense; i.e., these are not elements of the Hilbert space. Instead, we can define a qubit in the following way. We write $z_1 \in [-\frac{c}{2}, \frac{3c}{2})$ as $z_1 = \lfloor z_1 \rceil_c + \{z_1\}_c$ and regard $\lfloor z_1 \rceil_c / c$ as a bit. Then, we define

$$|0; z_1, z_2\rangle_{LS} \coloneqq |z_1, z_2\rangle_Z = \hat{V}(z_1, z_2)\, |\overline{0}\rangle\,, \tag{22}$$

$$|1; z_1, z_2\rangle_{LS} \coloneqq e^{ciz_2/2}\, |z_1 + c, z_2\rangle_Z = \hat{V}(z_1, z_2)\, |\overline{1}\rangle\,, \tag{23}$$

for $z_1, z_2 \in [-\frac{c}{2}, \frac{c}{2})$. In this way, we can decompose the Hilbert space $\mathcal{H}$ into a tensor product of a qubit Hilbert space $\mathbb{C}^2$ and an infinite-dimensional Hilbert space $\mathcal{H}_S$, i.e., $\mathcal{H} = \mathbb{C}^2 \otimes \mathcal{H}_S$, with

$$|\mu\rangle_L \otimes |z_1, z_2\rangle_S = |\mu; z_1, z_2\rangle_{LS}\,. \tag{24}$$

This decomposition is named the *stabilizer subsystem (SSS) decomposition* in Ref. [1]. This decomposition is analogous to decomposing the Hilbert space of physical qubits for a multi-qubit stabilizer code into a tensor product of the logical qubit and the syndrome subsystem, as discussed in Methods. Thus, we call the system L with the Hilbert space $\mathbb{C}^2$ in the decomposition the logical qubit and the system S with the Hilbert space $\mathcal{H}_S$ the syndrome subsystem. Note that this is a sub*system* decomposition of a single mode [1, 8], with the logical qubit being in a tensor product with the syndrome subsystem. This differs from typical approaches to error correction, which focus on ('logical' and 'error') sub*spaces* of a multi-qubit Hilbert space. We emphasize our approach by the use of the term 'subsystem' to identify the Hilbert spaces involved.

In the following, we will use the notation on both sides of Eq. (24) interchangeably. The quasi-periodic conditions (15) and (16) lead us to obtain

$$|\mu; z_1 + c, z_2\rangle_{LS} = e^{-ciz_2/2}\, |\mu \oplus 1; z_1, z_2\rangle_{LS}\,, \tag{25}$$

$$|\mu; z_1, z_2 + c\rangle_{LS} = e^{ciz_1/2}(-1)^\mu\, |\mu; z_1, z_2\rangle_{LS}\,, \tag{26}$$

where $\oplus$ denotes the summation modulo 2. The orthogonality relation (20) and the completeness relation (21) now become

$$\langle \mu; z_1, z_2 | \nu; z_1', z_2'\rangle_{LS} = \delta_{\mu\nu}\, \delta^{(c)}(z_1 - z_1')\, \delta^{(c)}(z_2 - z_2'), \tag{27}$$

$$\sum_{\mu=0,1} \int_{-\frac{c}{2}}^{\frac{c}{2}} dz_1 \int_{-\frac{c}{2}}^{\frac{c}{2}} dz_2 \, |\mu; z_1, z_2\rangle\langle \mu; z_1, z_2|_{LS} = \hat{I}. \tag{28}$$

In the following, we sometimes use a notation $|\psi; z_1, z_2\rangle$ with a two-dimensional complex vector $|\psi\rangle = \alpha\,|0\rangle + \beta\,|1\rangle$ to denote

$$|\psi; z_1, z_2\rangle_{LS} \coloneqq \alpha\, |0; z_1, z_2\rangle_{LS} + \beta\, |1; z_1, z_2\rangle_{LS} = |\psi\rangle_L \otimes |z_1, z_2\rangle_S = \hat{V}(z_1, z_2)\, |\overline{\psi}\rangle\,, \tag{29}$$

where $|\overline{\psi}\rangle$ denotes an ideal GKP state.

This representation developed in Ref. [1] has favorable properties. In particular, important Gaussian operations are concisely represented in this representation. Let $\hat{z}_1$ and $\hat{z}_2$ be operators satisfying

$$\hat{z}_1\, |z_1, z_2\rangle_S = z_1\, |z_1, z_2\rangle_S\,, \tag{30}$$

$$\hat{z}_2\, |z_1, z_2\rangle_S = z_2\, |z_1, z_2\rangle_S\,, \tag{31}$$

which implies $[\hat{z}_1, \hat{z}_2] = 0$. Formally, $\hat{z}_1$ and $\hat{z}_2$ correspond to $\{\hat{q}\}_c$ and $\{\hat{p}\}_c$, respectively, in the ordinary Hilbert space. Then, the stabilizer generators $\hat{S}_1$ and $\hat{S}_2$ defined in Eqs. (4) and (5) are represented in the SSS decomposition as

$$\hat{S}_1 = \hat{I}_L \otimes e_S^{-2ci\hat{z}_2}, \tag{32}$$

$$\hat{S}_2 = \hat{I}_L \otimes e_S^{2ci\hat{z}_1}. \tag{33}$$

Similarly, for the GKP logical operators $\overline{X}$ and $\overline{Z}$, we have

$$\overline{X} = \hat{X}_L \otimes e_S^{-ci\hat{z}_2}, \tag{34}$$

$$\overline{Z} = \hat{Z}_L \otimes e_S^{ci\hat{z}_1}, \tag{35}$$

where $\hat{X}$ and $\hat{Z}$ are the qubit Pauli operators. For the Fourier gate $\hat{F}$ defined in Eq. (9), we have [1]

$$\hat{F} = \hat{H}_L \otimes \hat{F}'_S, \tag{36}$$

where $\hat{H}$ is the qubit Hadamard gate and $\hat{F}'$ is defined to satisfy

$$\hat{F}' \left|z_1, z_2\right\rangle_S = \left|-z_2, z_1\right\rangle_S. \tag{37}$$

General Gaussian unitaries nontrivially couple the logical qubit and the syndrome subsystem. For example, the unitary $\exp(i\hat{q}^2/2)$ that works as the phase gate $\overline{S}$ for a GKP-encoded state is represented as

$$\exp(i\hat{q}^2/2)\left|\mu; z_1, z_2\right\rangle_{LS} = i^\mu \left|\mu; z_1, z_1 + z_2\right\rangle_{LS} \tag{38}$$

$$= \begin{cases} (-i)^\mu e^{-ciz_1/2} \left|\mu; z_1, z_1 + z_2 + c\right\rangle_{LS} & z_1 + z_2 < -c/2, \\ i^\mu \left|\mu; z_1, z_1 + z_2\right\rangle_{LS} & -c/2 \le z_1 + z_2 < c/2, \\ (-i)^\mu e^{ciz_1/2} \left|\mu; z_1, z_1 + z_2 - c\right\rangle_{LS} & c/2 \le z_1 + z_2, \end{cases} \tag{39}$$

where we used Eq. (16). Another example is the SUM gate defined in Eq. (12), which is represented as

$$\exp(-i\hat{q}_j\hat{p}_k)\left|\mu; z_1, z_2\right\rangle_{LS,j}\left|\nu; z'_1, z'_2\right\rangle_{LS,k} = \left|\mu; z_1, z_2 - z'_2\right\rangle_{LS,j}\left|\mu \oplus \nu; z'_1 + z_1, z'_2\right\rangle_{LS,k}, \tag{40}$$

where we forego writing down each case explicitly this time and leave interested readers to derive them using Eqs. (15) and (16) (see also Ref. [1]). In this way, general Gaussian unitaries do not act as a tensor product of operators on the logical qubit and the syndrome subsystem but rather entangle the two subsystems.

Let us finally observe how the POVM $\left|x\right\rangle\left\langle x\right|_q dx$ of the homodyne detection can be represented in the Zak basis and the stabilizer subsystem decomposition. First, we observe that

$$\left|x\right\rangle_q = \int_{-\frac{c}{2}}^{\frac{3c}{2}} dz_1 \int_{-\frac{c}{2}}^{\frac{c}{2}} dz_2 \left|z_1, z_2\right\rangle\left\langle z_1, z_2\right|_Z \left|x\right\rangle_q \tag{41}$$

$$= \int_{-\frac{c}{2}}^{\frac{3c}{2}} dz_1 \int_{-\frac{c}{2}}^{\frac{c}{2}} dz_2 \left|z_1, z_2\right\rangle_Z \frac{e^{-iz_1z_2/2}}{\sqrt{c}} \sum_{s\in\mathbb{Z}} e^{-2ciz_2 s}\delta(x - z_1 - 2cs) \tag{42}$$

$$= \int_{-\frac{c}{2}}^{\frac{c}{2}} dz_2 \frac{e^{-iz_2(x+2c\bar{s}(x))/2}}{\sqrt{c}} \left|x - 2c\bar{s}(x), z_2\right\rangle_Z, \tag{43}$$

where $\bar{s}(x)$ is a unique integer that satisfies $-\frac{c}{2} \le x - 2c\bar{s}(x) < \frac{3c}{2}$. Thus, the homodyne POVM can be written in the Zak basis as

$$\left|x\right\rangle\left\langle x\right|_q dx = \iint_{-\frac{c}{2}}^{\frac{c}{2}} \frac{e^{-i(z_2 - z'_2)(x+2c\bar{s}(x))/2}}{c} \left|x - 2c\bar{s}(x), z_2\right\rangle\left\langle x - 2c\bar{s}(x), z'_2\right|_Z dz_2 dz'_2 dx. \tag{44}$$

For example, a homodyne POVM element for the interval $\left[2cm + \Delta_1, 2cm + \Delta_2\right)$ with an integer m and $-\frac{c}{2} \le \Delta_1 < \Delta_2 \le \frac{3c}{2}$ is given by

$$\int_{2cm+\Delta_1}^{2cm+\Delta_2} \left|x\right\rangle\left\langle x\right|_q dx = \int_{\Delta_1}^{\Delta_2} dz_1 \iint_{-\frac{c}{2}}^{\frac{c}{2}} dz_2 dz'_2 \frac{e^{-i(z_2 - z'_2)(z_1+4cm)/2}}{c} \left|z_1, z_2\right\rangle\left\langle z_1, z'_2\right|_Z, \tag{45}$$

where $z_1 = x - 2cm$ and we used $\bar{s}(x) = m$. If we further sum up the above over all the integers m, we have

$$\sum_{m=-\infty}^{\infty} \int_{2cm+\Delta_1}^{2cm+\Delta_2} \left|x\right\rangle\left\langle x\right|_q dx = \int_{\Delta_1}^{\Delta_2} dz_1 \iint_{-\frac{c}{2}}^{\frac{c}{2}} dz_2 dz'_2 \frac{e^{-i(z_2 - z'_2)z_1/2}}{c} \sum_{n=-\infty}^{\infty} \delta\big((z_2 - z'_2)/c - n\big) \left|z_1, z_2\right\rangle\left\langle z_1, z'_2\right|_Z \tag{46}$$

$$= \int_{\Delta_1}^{\Delta_2} dz_1 \int_{-\frac{c}{2}}^{\frac{c}{2}} dz_2 \left|z_1, z_2\right\rangle\left\langle z_1, z_2\right|_Z \tag{47}$$

where we used the Poisson summation formula $\sum_{m=-\infty}^{\infty} e^{-2\pi i x m} = \sum_{n=-\infty}^{\infty} \delta(x-n)$ in the first equality and $-c < z_2 - z_2' < c$ in the second equality. When $\Delta_1 = -\frac{c}{2}$ and $\Delta_2 = \frac{c}{2}$, the measurement operator in the left-hand side of Eq. (47) corresponds to the event in which homodyne detection is performed, the outcome is binned to an integer multiple of $\sqrt{\pi}$, and the resulting integer is even. This is nothing but the logical $|\overline{0}\rangle$ event of the Pauli-Z measurement on the GKP code. In fact, Eq. (47) can be rewritten in this case as

$$\sum_{m=-\infty}^{\infty} \int_{2cm-\frac{c}{2}}^{2cm+\frac{c}{2}} |x\rangle\langle x|_q \, dx = |\overline{0}\rangle\langle\overline{0}|_L \otimes \iint_{-\frac{c}{2}}^{\frac{c}{2}} dz_1 dz_2 \, |z_1, z_2\rangle\langle z_1, z_2|_S = |\overline{0}\rangle\langle\overline{0}|_L \otimes \hat{I}_S. \tag{48}$$

The same applies to the case $\Delta_1 = \frac{c}{2}$ and $\Delta_2 = \frac{3c}{2}$, which corresponds to the GKP logical $|\overline{1}\rangle$ measurement.

B. Physical operations to implement a continuous-variable quantum computation with the GKP code

A fault-tolerant protocol aims to simulate an original circuit C on qubits (representing quantum computation) within any given target error $\varepsilon > 0$, i.e., to output a bit string sampled from a probability distribution close to the output probability distribution of the original circuit within error in the total variation distance at most ε [13, 14]. In this paper, we consider a CV fault-tolerant protocol achieving this simulation using a fault-tolerant circuit C' on CV systems with noise occurring according to a noise model specified later in Supplementary Note 1 D. We say that we can achieve fault-tolerant quantum computation if, for any $\varepsilon > 0$ and any original circuit C, we can construct a fault-tolerant circuit C' achieving this simulation under the noise model.

Here, we assume that the original circuit C is composed of computational-basis qubit-state preparations, single- and two-qubit gate operations including waits, and computational-basis measurements, where the universal set of gates includes the Pauli X and Z, the Hadamard H, the phase S, the Controlled-NOT CNOT, and the T gates, which are explicitly given as follows:

$$X = \begin{pmatrix} 0 & 1 \\ 1 & 0 \end{pmatrix}, \quad Z = \begin{pmatrix} 1 & 0 \\ 0 & -1 \end{pmatrix}, \quad H = \frac{1}{\sqrt{2}} \begin{pmatrix} 1 & 1 \\ 1 & -1 \end{pmatrix}, \quad S = \begin{pmatrix} 1 & 0 \\ 0 & i \end{pmatrix}, \quad \mathrm{CNOT} = \begin{pmatrix} 1 & 0 & 0 & 0 \\ 0 & 1 & 0 & 0 \\ 0 & 0 & 0 & 1 \\ 0 & 0 & 1 & 0 \end{pmatrix}, \quad T = \begin{pmatrix} 1 & 0 \\ 0 & e^{\frac{\pi i}{4}} \end{pmatrix}. \tag{49}$$

Note that the Pauli X and Z gates are auxiliary since they can be generated by combinations of the H and S gates, and the S gate as well due to $S = T^2$, but we add them to the list for convenience. The wait operation is to perform the identity gate

$$I = \begin{pmatrix} 1 & 0 \\ 0 & 1 \end{pmatrix}. \tag{50}$$

We call such circuit constituents (state preparations, gates, waits, and measurements) *locations*.

To simulate the original (qubit) circuit with CV systems, we use CV state preparations, gates, and measurements described in the previous section. However, we need to avoid the use of unphysical states such as the ideal GKP codeword in Eq. (1). Based on the subsystem decomposition explained in the previous section, we will introduce a family of physically realizable "approximate" GKP states as follows.

Definition 1 (s-parameterized GKP state)**.** For any qubit state $|\psi\rangle = \alpha\,|0\rangle + \beta\,|1\rangle$, we define an s-parameterized GKP state $\hat{\rho}_\psi^s$ as

$$\hat{\rho}_\psi^s \coloneqq \sum_i p_i \, |\rho_i^s[\psi]\rangle\langle\rho_i^s[\psi]| \,, \tag{51}$$

$$|\rho_i^s[\psi]\rangle \coloneqq \int_{-\infty}^{\infty} dz_1 \int_{-\infty}^{\infty} dz_2 \; f_i^s(z_1, z_2) \hat{V}(z_1, z_2) \, |\overline{\psi}\rangle \,, \tag{52}$$

where p_i $(i = 1, 2, \ldots)$ denotes probabilities summed up to one, $|\overline{\psi}\rangle = \alpha\,|\overline{0}\rangle + \beta\,|\overline{1}\rangle$ denotes an ideal GKP state, and $f_i^s(x, y)$ denotes a function of (x, y) that has a support only in $[-s, s) \times [-s, s)$ and satisfies $\langle\rho_i^s[\psi]|\rho_i^s[\psi]\rangle = 1$. Notice that the notation $\hat{\rho}_\psi^s$ leaves implicit the mixing probabilities $\{p_i\}$ and the s-supported functions $\{f_i^s\}$.

From this definition and Eqs. (22) and (23), a state $|\rho_i^s[\psi]\rangle$ has the form

$$|\rho_i^s[\psi]\rangle = |\psi\rangle_L \otimes \int_{-\frac{c}{2}}^{\frac{c}{2}} dz_1 \int_{-\frac{c}{2}}^{\frac{c}{2}} dz_2 \; f_i^s(z_1, z_2) \, |z_1, z_2\rangle_S \tag{53}$$

when $s < c/2$. Thus, it can be regarded as an "approximate GKP codeword". In contrast to the typical definition of approximate GKP states [3, 15], the state $\hat{\rho}^s_{\psi}$ has *exactly* the same logical content as the ideal one $|\overline{\psi}\rangle$ as long as $s < c/2$.

Having defined the class of physically realizable approximate GKP states that we will consider, we can now list physical operations to implement our CV quantum circuit:

- preparation of a state from a finite set $\mathcal{S}$ including $\hat{\rho}^s_0$, $\hat{\rho}^s_Y$, and $\hat{\rho}^s_{\frac{\pi}{8}}$ with s chosen to be sufficiently smaller than $c/2$, which are defined through Eq. (51) with $|Y\rangle = (|0\rangle + i\,|1\rangle)/\sqrt{2}$ and $|\frac{\pi}{8}\rangle = (|0\rangle + e^{\pi i/4}\,|1\rangle)/\sqrt{2}$,
- displacement $\hat{V}(v_1, v_2)$ by an amount drawn from a finite set V of possible pairs (v_1, v_2) of values, including $(c, 0)$ and $(0, c)$,
- phase rotation $\hat{R}(\theta)$ by an angle drawn from a finite set Θ for possible values of θ, including $\pi/2$,
- SUM gate $\exp(-i\hat{q}_1\hat{p}_2)$,
- q- and p-homodyne detection,
- waiting (or using delay lines).

Later, it will be clear that to avoid a logical error, the parameter s in a prepared state should be smaller than $c/(2N_{\text{2-mode}})$ with $N_{\text{2-mode}}$ denoting the maximum number of two-mode gates that a prepared state goes through before a measurement. Note that if we adopt the Knill-type error correction [16], which we will do, $N_{\text{2-mode}}$ is a constant number.

C. Energy-constrained diamond norm

In this section, we introduce a distance measure between CV quantum channels that we use in the next section. The distance measure is necessary to define a noise strength for a given noise model; i.e., small noise strength implies closeness to the identity channel. However, there are subtleties in the distance measure for channels in infinite-dimensional systems, as discussed in the main text with an example of a phase-rotation channel $\mathcal{R}[\theta]$. Our physical intuition that $\mathcal{R}[\theta]$ is close to the identity map when $\theta > 0$ is very close to 0 comes from the fact that quantum states we usually generate and treat in a lab are not very susceptible to a small phase rotation, whereas the diamond-norm distance tells us it is far from the identity map since a coherent state with the amplitude α distinguishes these two channels better and better as $|\alpha| \to \infty$. This raises the following two issues to discuss. On one hand, if a quantum state during quantum computation is "reasonably good", then noise such as an infinitesimal rotation should be well approximated by the identity map. On the other hand, for this to be true, we need to take care that a quantum state during computation is ensured to be kept "reasonably good". We postpone the discussion of the second issue and focus on the first issue here.

In this paper, quantum states with the energy constraint are regarded as "reasonably good" quantum states, which seems reasonable in terms of an actual experiment. More precisely, we regard the following set of quantum states as those realizable in an experiment and thus in a CV quantum circuit.

Definition 2 (The set of energy-constrained quantum states)**.** Given an energy bound $E > 0$ on a system (or equivalently, a mode) Q, the set $\mathfrak{S}_E(\mathcal{H}_Q)$ of energy-constrained density operators on the system Q is defined as

$$\mathfrak{S}_E(\mathcal{H}_Q) = \{\hat{\rho} \in \mathcal{B}(\mathcal{H}_Q) : \hat{\rho} \geq 0, \mathrm{Tr}(\hat{\rho}) \leq 1, \mathrm{Tr}(\hat{n}\hat{\rho}) \leq E\,\mathrm{Tr}(\hat{\rho})\}. \tag{54}$$

The set of all density operators without energy constraint is simply denoted by $\mathfrak{S}(\mathcal{H}_Q)$.

In the definition above, as well as in the following discussions in this section, we identify the number operator $\hat{n}$ as a Hamiltonian of a system, with the optical system in mind, but the same argument holds for any Hamiltonian operator $\hat{H}$ that satisfies the Gibbs hypothesis, i.e., $\mathrm{Tr}[\exp(-\beta\hat{H})] < \infty$ for all $\beta > 0$ [17, 18]. Note that the number operator $\hat{n}$ clearly satisfies this condition. A particularly nice property of the set $\mathfrak{S}_E(\mathcal{H}_Q)$ for a finite E is that it is compact with respect to the trace norm [19], while $\mathfrak{S}(\mathcal{H}_Q)$ is not. For this set of energy-constrained quantum states, we can define the energy-constrained diamond norm considered in Refs. [17–24].

Definition 3 (Energy-constrained diamond norm [23])**.** Let Φ be a Hermitian-preserving linear map (not necessarily completely positive or even positive) acting on operators on $\mathcal{H}_Q$. For $E > 0$, the energy-constrained diamond norm $\|\Phi\|^E_\diamond$ of Φ is defined as

$$\|\Phi\|^E_\diamond := \sup_{\hat{\rho}_{RQ} \in \mathfrak{S}(\mathcal{H}_{RQ}) : \hat{\rho}_Q \in \mathfrak{S}_E(\mathcal{H}_Q)} \|\mathrm{Id}_R \otimes \Phi(\hat{\rho}_{RQ})\|_1, \tag{55}$$

where Id_R denotes the identity map of operators on $\mathcal{H}_R \cong \mathcal{H}_Q$, $\hat{\rho}_Q := \mathrm{Tr}_R[\hat{\rho}_{RQ}]$, and $\|\hat{T}\|_1 := \mathrm{Tr}\sqrt{\hat{T}^\dagger \hat{T}}$. We let $\|\Phi\|_\diamond$ denote the diamond norm without energy constraint, i.e.,

$$\|\Phi\|_\diamond := \sup_{\hat{\rho}_{RQ} \in \mathfrak{S}(\mathcal{H}_{RQ})} \|\mathrm{Id}_R \otimes \Phi(\hat{\rho}_{RQ})\|_1 = \sup_{\hat{T}_{RQ} \in \mathcal{B}(\mathcal{H}_{RQ}) : \|\hat{T}_{RQ}\|_1 \leq 1} \|\mathrm{Id}_R \otimes \Phi(\hat{T}_{RQ})\|_1. \tag{56}$$

For a composite system Q_1Q_2 with the energy constraint E_1 on Q_1 and E_2 on Q_2, we define $\|\Phi_{Q_1Q_2}\|_\diamond^{E_1,E_2}$ as

$$\|\Phi_{Q_1Q_2}\|_\diamond^{E_1,E_2} := \sup_{\hat{\rho}_{RQ_1Q_2} \in \mathfrak{S}(\mathcal{H}_{RQ_1Q_2}) : \hat{\rho}_{Q_1} \in \mathfrak{S}_{E_1}(\mathcal{H}_{Q_1}), \hat{\rho}_{Q_2} \in \mathfrak{S}_{E_2}(\mathcal{H}_{Q_2})} \|\mathrm{Id}_R \otimes \Phi_{Q_1Q_2}(\hat{\rho}_{RQ_1Q_2})\|_1, \tag{57}$$

where $\mathcal{H}_R \cong \mathcal{H}_{Q_1Q_2}$.

This norm defines a distance between quantum channels called the energy-constrained diamond-norm distance. Many favorable properties of the diamond norm in the finite-dimensional case are recovered in the energy-constrained diamond norm [17, 23]. We cite some of the properties that will be used later.

- (Monotonicity under energy increase) For $0 < E_1 \leq E_2$, the energy-constrained diamond norm satisfies

$$\|\Phi\|_\diamond^{E_1} \leq \|\Phi\|_\diamond^{E_2}. \tag{58}$$

  In particular, $\|\Phi\|_\diamond^E \leq \|\Phi\|_\diamond$ holds for any $E > 0$.

- (Submultiplicativity under composition) Let Φ be a Hermitian-preserving linear map and $\Psi : \mathfrak{S}_{E_1}(\mathcal{H}_Q) \to \mathfrak{S}_{E_2}(\mathcal{H}_Q)$ be a completely positive map. Then, we have

$$\|\Phi \circ \Psi\|_\diamond^{E_1} \leq \|\Phi\|_\diamond^{E_2} \|\Psi\|_\diamond^{E_1}. \tag{59}$$

  Furthermore, for another Hermitian-preserving linear map $\Psi' : \mathfrak{S}_{E_1}(\mathcal{H}_Q) \to \mathcal{B}(\mathcal{H}_Q)$, we have

$$\|\Phi \circ \Psi'\|_\diamond^{E_1} \leq \|\Phi\|_\diamond \|\Psi'\|_\diamond^{E_1}. \tag{60}$$

- (Supermultiplicativity under tensor product) For Hermitian-preserving linear maps Φ_{Q_1} and Φ_{Q_2} of operators on $\mathcal{H}_{Q_1}$ and $\mathcal{H}_{Q_1}$, respectively, we have

$$\|\Phi_{Q_1} \otimes \Phi_{Q_2}\|_\diamond^{E_1,E_2} \geq \|\Phi_{Q_1}\|_\diamond^{E_1} \|\Phi_{Q_2}\|_\diamond^{E_2}. \tag{61}$$

  The equality holds when at least one of Φ_{Q_1} and Φ_{Q_2} is completely positive. This property can straightforwardly be derived from the facts that $\|\hat{A} \otimes \hat{B}\|_1 = \|\hat{A}\|_1 \|\hat{B}\|_1$ holds and that there exists a state $\hat{\rho}_{RQ_1Q_2}$ on the composite system RQ_1Q_2 such that $\hat{\rho}_{Q_1Q_2}$ is an entangled state and $\hat{\rho}_{Q_i} \in \mathfrak{S}_{E_i}(\mathcal{H}_{Q_i})$ for $i = 1, 2$.

- (Achievability) From Lemma 1 of Ref. [25] and Corollary 6 of Ref. [20], the optimization involved in the defining expression 55 of the energy-constrained diamond norm can be taken over a compact convex subset of $\mathfrak{S}(\mathcal{H}_{RQ})$ that is compatible with $\mathfrak{S}_E(\mathcal{H}_Q)$. Thus, there always exists $\hat{\rho}_{QR}$ with $\hat{\rho}_Q \in \mathfrak{S}_E(\mathcal{H}_Q)$ such that $\|\Phi_Q\|_\diamond^E = \|\mathrm{Id}_R \otimes \Phi_Q(\hat{\rho}_{RQ})\|_1$.

One shortcoming of this norm is that one needs extra care when one applies it to a composite map, which can already be seen in the preconditions above. It is particularly because $(\hat{\rho}_A - \hat{\sigma}_A)_\pm \notin \mathfrak{S}_E(\mathcal{H}_A)$ even though $\hat{\rho}_A, \hat{\sigma}_A \in \mathfrak{S}_E(\mathcal{H}_A)$, where $(\hat{X})_\pm := \hat{X} \pm |\hat{X}|$ is the (subnormalized) density operator corresponding to the positive part (and the negative part, respectively) of $\hat{X}$. However, by appropriately enlarging an energy-constrained set, we have a bound on the energy-constrained diamond norm of a composite map with respective norms, as we show in the following proposition by generalizing the arguments used for Lemma 1 and Theorem 1 in Ref. [26].

Proposition 4 (Norm of a map acting on the difference of energy-constrained density operators)**.** Given $E > 0$, let $\hat{\rho}_{AR}$ and $\hat{\sigma}_{AR}$ be density operators with $\hat{\rho}_A, \hat{\sigma}_A \in \mathfrak{S}_E(\mathcal{H}_A)$ and $\|\hat{\rho}_{AR} - \hat{\sigma}_{AR}\|_1 \leq 2\epsilon$ with $0 < \epsilon < 1$. Then, for any Hermitian-preserving linear map Φ_A on the system A, we have

$$\|\Phi_A \otimes \mathrm{Id}_R(\hat{\rho}_{AR} - \hat{\sigma}_{AR})\|_1 \leq 10\epsilon \|\Phi_A\|_\diamond^{E/\epsilon^2}. \tag{62}$$

To prove Proposition 4, we use the following auxiliary lemma, which is a reformulation of what is proved in Lemma 1 in Ref. [26]. We provide the proof of this auxiliary lemma for completeness of our analysis.

Lemma 5 (Finite-dimensional approximation of energy-constrained density operators: Footnote 5 in the proof of Lemma 1 in Ref. [26], Lemma 3 of Ref. [18]). Given $E>0$, let $\hat{\rho}_{AR}$ be a density operator with $\hat{\rho}_A \in \mathfrak{S}_E(\mathcal{H}_A)$. Then, for any purification $|\phi\rangle_{ARS}$ of $\hat{\rho}_{AR}$, there exist a pure state $|\tilde{\phi}\rangle_{ARS}$ and an error parameter $t\in(0,1)$ such that

$$\mathrm{supp}(\tilde{\phi}_A) \subset \mathrm{span}\{|0\rangle_A, \ldots, |\lfloor E/t^2 \rfloor - 1\rangle_A\}, \tag{63}$$

$$\frac{1}{2}\| \, |\phi\rangle\langle\phi| - |\tilde{\phi}\rangle\langle\tilde{\phi}| \, \|_1 \le t, \tag{64}$$

$$|\phi\rangle\langle\phi| - |\tilde{\phi}\rangle\langle\tilde{\phi}| = t\,|\gamma_+\rangle\langle\gamma_+| - t\,|\gamma_-\rangle\langle\gamma_-| \,, \tag{65}$$

$$\langle\gamma_\pm|\gamma_\pm\rangle = 1, \quad \langle\gamma_+|\gamma_-\rangle = 0, \tag{66}$$

$$\mathrm{Tr}[\hat{n}_A(\mathrm{Tr}_{RS}[|\gamma_\pm\rangle\langle\gamma_\pm|_{ARS}])] \le E/t^2, \tag{67}$$

where $\tilde{\phi}_A := \mathrm{Tr}_{RS}[|\tilde{\phi}\rangle\langle\tilde{\phi}|_{ARS}]$, $\mathrm{supp}(\tilde{\phi}_A)$ is the support of $\tilde{\phi}_A$, $\{|n\rangle_A\}_{n=0}^\infty$ is the Fock basis of $\mathcal{H}_A$, and $\hat{n}_A = \sum_{n=0}^\infty n\,|n\rangle\,\langle n|_A$ is the number operator (i.e. the Hamiltonian of the system A).

Proof. Let $|\phi\rangle_{ARS}$ be a purification of $\hat{\rho}_{AR}$. We have, without loss of generality,

$$|\phi\rangle_{ARS} = \sum_{j,k=0}^{\infty} \sqrt{p_{jk}} e^{i\theta_{jk}} \, |j\rangle_A \, |\alpha_k\rangle_{RS} \,, \tag{68}$$

where $|j\rangle_A$ denotes the Fock state, $\{|\alpha_k\rangle\}_{k=0}^\infty$ is an orthonormal system in $\mathcal{H}_R \otimes \mathcal{H}_S$, and $\{p_{jk}\}_{j,k=0}^\infty$ are probabilities satisfying $\sum_{j,k=0}^\infty p_{jk} = 1$. Let $d > E$ be any integer and define δ_d as $\delta_d := \sum_{j\ge d} \tilde{p}_j$, where $\tilde{p}_j := \sum_{k=0}^\infty p_{jk}$. We further define $|\tilde{\phi}\rangle_{ARS}$ as

$$|\tilde{\phi}\rangle_{ARS} := (1-\delta_d)^{-1/2} \sum_{j=0}^{d-1} \sum_{k=0}^{\infty} \sqrt{p_{jk}} e^{i\theta_{jk}} \, |j\rangle_A \, |\alpha_k\rangle_{RS} \,. \tag{69}$$

From the energy constraint, we have

$$\delta_d d \le \sum_{j\ge d} \sum_{k=0}^{\infty} p_{jk} \, \langle j|_A \, \langle\alpha_k|_{RS} \, \hat{n}_A \otimes \hat{I}_{RS} \, |j\rangle_A \, |\alpha_k\rangle_{RS} \le E, \tag{70}$$

and thus we have $\delta_d \le E/d < 1$. Since $\langle\phi|\tilde{\phi}\rangle = (1-\delta_d)^{1/2}$, we have

$$\| \, |\phi\rangle\langle\phi| - |\tilde{\phi}\rangle\langle\tilde{\phi}| \, \|_1 = 2\sqrt{1 - |\, \langle\phi|\tilde{\phi}\rangle \,|^2} = 2\sqrt{\delta_d}, \tag{71}$$

where we used the well-known relation between the trace distance and the fidelity for pure states in the first equality. From the diagonalization of the 2×2 matrix $|\phi\rangle\langle\phi|_{ARS} - |\tilde{\phi}\rangle\langle\tilde{\phi}|_{ARS}$, we have

$$|\phi\rangle\langle\phi|_{ARS} - |\tilde{\phi}\rangle\langle\tilde{\phi}|_{ARS} = \sqrt{\delta_d}\, |\gamma_+\rangle\langle\gamma_+|_{ARS} - \sqrt{\delta_d}\, |\gamma_-\rangle\langle\gamma_-|_{ARS} \,, \tag{72}$$

where $|\gamma_\pm\rangle$ are normalized eigenvectors of positive and negative parts, respectively, with $\langle\gamma_+|\gamma_-\rangle = 0$. Written explicitly, these eigenvectors are

$$|\gamma_\pm\rangle_{ARS} = p_\pm \, |\phi\rangle_{ARS} + q_\pm \, |\tilde{\phi}\rangle_{ARS} \,, \tag{73}$$

with $p_\pm = \sqrt{(1\pm\sqrt{\delta_d})/(2\delta_d)}$ and $q_\pm = -\sqrt{(1\mp\sqrt{\delta_d})/(2\delta_d)}$.

Now, we would like to bound the average energy of $|\gamma_\pm\rangle_{ARS}$ in the system A. We have

$$\begin{aligned}
\langle\gamma_\pm| \, \hat{n}_A \otimes \hat{I}_{RS} \, |\gamma_\pm\rangle &= p_\pm^2 \, \langle\phi| \, \hat{n}_A \otimes \hat{I}_{RS} \, |\phi\rangle + q_\pm^2 \, \langle\tilde{\phi}| \, \hat{n}_A \otimes \hat{I}_{RS} \, |\tilde{\phi}\rangle + 2p_\pm q_\pm \Re(\langle\phi| \, \hat{n}_A \otimes \hat{I}_{RS} \, |\tilde{\phi}\rangle) &(74)\\
&= p_\pm^2 \sum_{j=0}^{\infty} \tilde{p}_j j + q_\pm^2 (1-\delta_d)^{-1} \sum_{k=0}^{d-1} \tilde{p}_k k + 2p_\pm q_\pm (1-\delta_d)^{-1/2} \sum_{l=0}^{d-1} \tilde{p}_l l &(75)\\
&= [p_\pm^2 + 2p_\pm q_\pm (1-\delta_d)^{-1/2} + q_\pm^2 (1-\delta_d)^{-1}] T + p_\pm^2 U, &(76)
\end{aligned}$$

where $T := \sum_{j=0}^{d-1} \tilde{p}_j j$ and $U := \sum_{k\ge d} \tilde{p}_k k$. Using the explicit form of $p_\pm$ and $q_\pm$, we have

$$(76) = [p_\pm + q_\pm (1-\delta_d)^{-1/2}]^2 \, T + p_\pm^2 U \tag{77}$$

$$= \frac{1}{2\delta_d}\left[\left(\sqrt{1\pm\sqrt{\delta_d}} - \frac{1}{\sqrt{1\pm\sqrt{\delta_d}}}\right)^2 T + (1\pm\sqrt{\delta_d})U\right] \tag{78}$$

$$= \frac{1}{2\delta_d}\left[\frac{\delta_d}{1\pm\sqrt{\delta_d}}T + (1\pm\sqrt{\delta_d})U\right], \tag{79}$$

Applying the inequalities $T = \sum_{k=0}^{\infty}\tilde{p}_k\sum_{j=0}^{d-1}\tilde{p}_j j \le \sum_{j=0}^{d-1}\sum_{k=0}^{\infty}\tilde{p}_j\tilde{p}_k k = (1-\delta_d)E$ and $U \le E$ to the above, we have

$$\langle\gamma_\pm|\,\hat{n}_A\otimes\hat{I}_{RS}\,|\gamma_\pm\rangle \le \frac{1}{2\delta_d}[\delta_d(1\mp\sqrt{\delta_d})E + (1\pm\sqrt{\delta_d})E] \tag{80}$$

$$\le \frac{1}{2\delta_d}[(1\mp\sqrt{\delta_d})E + (1\pm\sqrt{\delta_d})E] = \frac{E}{\delta_d}, \tag{81}$$

where we used $\delta_d \le 1$. Let us replace $\delta_d \mapsto t^2$ with $t\in(0,1)$. Then, from Eq. (81) and $t^2 d \le E$, we have

$$\mathrm{Tr}_{RS}[|\gamma_\pm\rangle\langle\gamma_\pm|_{ARS}] \in \mathfrak{S}_{E/t^2}(\mathcal{H}_A), \tag{82}$$

$$|\tilde{\phi}\rangle_{ARS} \in (\hat{P}_A^{\lfloor E/t^2\rfloor}\otimes\hat{I}_{RS})\mathcal{H}_{ARS}, \tag{83}$$

where

$$\hat{P}^d \coloneqq \sum_{n=0}^{d-1}|n\rangle\langle n| \qquad \text{for integer } d. \tag{84}$$

Therefore, from Eqs. (71), (72), (82), and (83), we obtain the statement. Q.E.D.

Using Lemma 5, we prove Proposition 4 as follows.

Proof of Proposition 4. Let $|\phi\rangle_{ARS}$ be a purification of $\hat{\rho}_{AR}$. Using Lemma 5, we obtain a t-approximation $|\tilde{\phi}\rangle_{ARS}$ of $|\phi\rangle_{ARS}$. Then, for any Hermitian-preserving linear map Φ_A, we have

$$\|\Phi_A\otimes\mathrm{Id}_{RS}(|\phi\rangle\langle\phi|_{ARS} - |\tilde{\phi}\rangle\langle\tilde{\phi}|_{ARS})\|_1 = t\|\Phi_A\otimes\mathrm{Id}_{RS}(|\gamma_+\rangle\langle\gamma_+|_{ARS} - |\gamma_-\rangle\langle\gamma_-|_{ARS})\|_1 \tag{85}$$

$$\le t\|\Phi_A\otimes\mathrm{Id}_{RS}(|\gamma_+\rangle\langle\gamma_+|_{ARS})\|_1 + t\|\Phi_A\otimes\mathrm{Id}_{RS}(|\gamma_-\rangle\langle\gamma_-|_{ARS})\|_1 \tag{86}$$

$$\le 2t\|\Phi_A\|_\diamond^{E/t^2}, \tag{87}$$

where we used Eq. (65) of Lemma 5 in the first equality, the triangle inequality in the first inequality, and Eq. (67) of Lemma 5 with Eq. (55) in the last inequality.

An analogous derivation can be carried out for a purification $|\psi\rangle_{ARS}$ of $\hat{\sigma}_{AR}$. With a t-approximation $|\tilde{\psi}\rangle_{ARS}$ of $|\psi\rangle_{ARS}$, we obtain from Lemma 5:

$$\|\Phi_A\otimes\mathrm{Id}_{RS}(|\psi\rangle\langle\psi|_{ARS} - |\tilde{\psi}\rangle\langle\tilde{\psi}|_{ARS})\|_1 \le 2t\|\Phi_A\|_\diamond^{E/t^2}, \tag{88}$$

as in Eq. (87).

In the following, we will analyze an upper bound on $\|\Phi_A\otimes\mathrm{Id}_R(\hat{\rho}_{AR}-\hat{\sigma}_{AR})\|_1$. For this analysis, we define $\hat{\rho}'_{AR} \coloneqq \mathrm{Tr}_S[|\tilde{\phi}\rangle\langle\tilde{\phi}|_{ARS}]$ and $\hat{\sigma}'_{AR} \coloneqq \mathrm{Tr}_S[|\tilde{\psi}\rangle\langle\tilde{\psi}|_{ARS}]$. Importantly, the operator $\mathrm{Tr}_R[\hat{\rho}'_{AR}-\hat{\sigma}'_{AR}]$ has a finite support in $\mathrm{span}\{|0\rangle_A,\ldots,|\lfloor E/t^2\rfloor-1\rangle_A\}$ on A, which makes our analysis possible; to analyze bounds for this operator, we introduce a set of (Hermitian and unit-trace-norm) operators on $\mathcal{H}_A\otimes\mathcal{H}_R$ with a finite support on A, i.e.,

$$\mathcal{T}_{E/t^2} \coloneqq \left\{\hat{X}_{AR}\in\mathcal{B}\left((\hat{P}_A^{\lfloor E/t^2\rfloor}\otimes\hat{I}_R)\mathcal{H}_{AR}\right) : \hat{X}_{AR} = \hat{X}_{AR}^\dagger, \|\hat{X}_{AR}\|_1 = 1\right\}, \tag{89}$$

where $\mathcal{B}\left((\hat{P}_A^{\lfloor E/t^2\rfloor}\otimes\hat{I}_R)\mathcal{H}_{AR}\right)$ is the set of bounded operators on $(\hat{P}_A^{\lfloor E/t^2\rfloor}\otimes\hat{I}_R)\mathcal{H}_{AR}$. Then, we have the following (with further explanation given below):

$$\|\Phi_A\otimes\mathrm{Id}_R(\hat{\rho}_{AR}-\hat{\sigma}_{AR})\|_1 \tag{90}$$

$$\le \|\Phi_A\otimes\mathrm{Id}_R(\hat{\rho}_{AR}-\hat{\rho}'_{AR})\|_1 + \|\Phi_A\otimes\mathrm{Id}_R(\hat{\sigma}_{AR}-\hat{\sigma}'_{AR})\|_1 + \|\Phi_A\otimes\mathrm{Id}_R(\hat{\rho}'_{AR}-\hat{\sigma}'_{AR})\|_1 \tag{91}$$

$$\le \|\Phi_A\otimes\mathrm{Id}_{RS}(|\phi\rangle\langle\phi| - |\tilde{\phi}\rangle\langle\tilde{\phi}|)\|_1 + \|\Phi_A\otimes\mathrm{Id}_R(|\psi\rangle\langle\psi| - |\tilde{\psi}\rangle\langle\tilde{\psi}|)\|_1 + \sup_{\hat{X}_{AR}\in\mathcal{T}_{E/t^2}}\|\Phi_A\otimes\mathrm{Id}_R(\|\hat{\rho}'_{AR}-\hat{\sigma}'_{AR}\|_1\hat{X}_{AR})\|_1 \tag{92}$$

$$\leq 4t\|\Phi_A\|_\diamond^{E/t^2} + \sup_{\hat{X}_{AR}\in\mathcal{T}_{E/t^2}} \|\Phi_A\otimes\mathrm{Id}_R(\hat{X}_{AR})\|_1\|\hat{\rho}'_{AR}-\hat{\sigma}'_{AR}\|_1, \tag{93}$$

where the triangle inequality is used in the first inequality, the monotonicity of the trace distance under tracing out and the fact $\hat{\rho}'_{AR}, \hat{\sigma}'_{AR} \in \mathcal{T}_{E/t^2}$ are used in the second inequality, and Eqs. (87) and (88) are used in the last inequality. Since Φ_A is a Hermitian-preserving map, due to the convexity of the norm, we can replace maximization over $\hat{X}_{AR}$ in Eq. (93) with that over rank-1 projections $\{|\tau\rangle\langle\tau|_{AR}\}$, where $|\tau\rangle_{AR} \in (\hat{P}_A^{\lfloor E/t^2\rfloor}\otimes\hat{I}_R)\mathcal{H}_{AR}$ [27]. In other words, the optimization over Hermitian operators $\hat{X}_{AR}\in\mathcal{T}_{E/t^2}$ (not necessarily quantum states) can be replaced with that over (pure) quantum states $|\tau\rangle_{AR} \in (\hat{P}_A^{\lfloor E/t^2\rfloor}\otimes\hat{I}_R)\mathcal{H}_{AR}$ within a finite-dimensional subspace in the system A, for which the energy of A is well-defined and bounded by

$$\langle\tau|\,\hat{n}_A\otimes\hat{I}_R\,|\tau\rangle \leq E/t^2. \tag{94}$$

Therefore, we obtain

$$\sup_{\hat{X}_{AR}\in\mathcal{T}_{E/t^2}} \|\Phi_A\otimes\mathrm{Id}_R(\hat{X}_{AR})\|_1 \leq \sup_{|\tau\rangle\in(\hat{P}_A^{\lfloor E/t^2\rfloor}\otimes\hat{I}_R)\mathcal{H}_{AR}:\langle\tau|\tau\rangle=1} \|\Phi_A\otimes\mathrm{Id}_R(|\tau\rangle\langle\tau|_{AR})\|_1 \tag{95}$$

$$\leq \|\Phi_A\|_\diamond^{E/t^2}, \tag{96}$$

where the first inequality is this replacement, and the second inequality follows from the energy bound (94) and the definition (55) of the energy-constrained diamond norm. We also have

$$\|\hat{\rho}'_{AR}-\hat{\sigma}'_{AR}\|_1 \leq \|\hat{\rho}_{AR}-\hat{\sigma}_{AR}\|_1 + \|\hat{\rho}_{AR}-\hat{\rho}'_{AR}\|_1 + \|\hat{\sigma}_{AR}-\hat{\sigma}'_{AR}\|_1 \leq 2\epsilon + 4t, \tag{97}$$

where the first term of the right-most expression comes from the assumption of this proposition, and the second term comes from Eq. (71) and the monotonicity of the trace distance under tracing out applied to both $\|\hat{\rho}_{AR}-\hat{\rho}'_{AR}\|_1$ and $\|\hat{\sigma}_{AR}-\hat{\sigma}'_{AR}\|_1$. From Eqs. (93), (96), and (97), we prove the statement of the proposition by substituting $t=\epsilon$, so that the overall coefficient is $4t+2\epsilon+4t\big|_{t\to\epsilon} = 10\epsilon$. Q.E.D.

As an immediate corollary of the above theorem, we have the following generalization of the submultiplicativity property cited above.

Corollary 6. Let Φ_A be a Hermitian-preserving map and $\Psi_A, \tilde{\Psi}_A : \mathfrak{S}_{E_1}(\mathcal{H}_A) \to \mathfrak{S}_{E_2}(\mathcal{H}_A)$ be two CPTP maps such that $\|\Psi_A - \tilde{\Psi}_A\|_\diamond^{E_1} \leq 2\epsilon$. Then, we have

$$\|\Phi_A\circ(\Psi_A-\tilde{\Psi}_A)\|_\diamond^{E_1} \leq 10\epsilon\|\Phi_A\|_\diamond^{E_2/\epsilon^2}. \tag{98}$$

Proof. From the achievability mentioned above, we have a state $\hat{\tau}_{AR}$ with $\hat{\tau}_A \in \mathfrak{S}_{E_1}(\mathcal{H}_A)$ such that $\|\Phi_A\circ(\Psi_A - \tilde{\Psi}_A)\|_\diamond^{E_1} = \|[\Phi_A\circ(\Psi_A-\tilde{\Psi}_A)]\otimes\mathrm{Id}_R(\hat{\tau}_{AR})\|_1$. Then, we can apply Proposition 4 by substituting $\hat{\rho}_{AR} = \Psi_A\otimes\mathrm{Id}_R(\hat{\tau}_{AR})$ and $\hat{\sigma}_{AR} = \tilde{\Psi}_A\otimes\mathrm{Id}_R(\hat{\tau}_{AR})$ and using $\|\hat{\rho}_{AR}-\hat{\sigma}_{AR}\|_1 = \|(\Psi_A-\tilde{\Psi}_A)\otimes\mathrm{Id}_R(\hat{\tau}_{AR})\|_1 \leq \|\Psi_A-\tilde{\Psi}_A\|_\diamond^{E_1} \leq 2\epsilon$. Q.E.D.

D. Noise model

In the previous sections, we described the physical operations that implement a CV quantum circuit and introduced a distance measure between noise channels to evaluate the noise strength. In this section, we consider a noise model for these physical operations, which then leads to a noise model for each location of CV quantum computation. The noise model in this paper is an independent Markovian noise model, meaning that a noise at a given location can be described as a quantum channel that is independent of any other location in the quantum circuit. This noise model is general as long as computational modes do not strongly interact with each other (so that there is no correlated noise in space) and do not repeatedly interact with the same environmental mode (so that there is no correlated noise in time). These assumptions may be reasonable in propagating optical systems where the interaction between modes is weak and they move fast.

Before moving on to the formal definition of the noise model and its noise strength, we define the following channel.

Definition 7 (s-parameterized noise channel)**.** An s-parameterized noise channel $\mathcal{N}^s$ is defined as a noise channel whose Kraus operators are linear combinations of elements in $\{\hat{V}(z_1,z_2) : (z_1,z_2)\in[-s,s)\times[-s,s)\}$ for a single-mode gate, i.e.,

$$\mathcal{N}^s(\hat{\rho}) = \int_{\mathbb{R}^4} d^2z\, d^2z'\ \mathcal{E}_s(z_1,z_2,z'_1,z'_2)\hat{V}(z_1,z_2)\hat{\rho}\hat{V}(z'_1,z'_2)^\dagger, \tag{99}$$

where the function $\mathcal{E}_s(z_1, z_2, z_1', z_2')$ is supported only on the Cartesian product $\prod_{i=1}^4 [-s, s)$. Likewise, for a two-mode gate, the Kraus operators are linear combinations of elements in $\{\hat{V}(z_1, z_2) \otimes \hat{V}(z_1', z_2') : (z_1, z_2) \in [-s, s) \times [-s, s), (z_1', z_2') \in [-s, s) \times [-s, s)\}$.

Now, we define the noise models against which we prove fault tolerance in this paper.

Definition 8 ((s, ϵ)-independent Markovian noise model for preparation)**.** Given a constant $E_{\rm prep} > 0$, consider a collection $\mathcal{C}'$ of physical systems that comprise a CV quantum computer, and regard other physical systems as environments. A noisy physical state preparation for a GKP logical state $|\overline{\psi}\rangle\langle\overline{\psi}|$ is said to obey an (s, ϵ)-independent Markovian noise model if it prepares a noisy state $\hat{\rho}_\psi^{\rm noisy} \in \mathfrak{S}_{E_{\rm prep}}(\mathcal{H})$, independent of other physical systems in $\mathcal{C}'$, such that there exists a state $\hat{\rho}_\psi^s$ defined in Def. 1 that satisfies $\hat{\rho}_\psi^s \in \mathfrak{S}_{E_{\rm prep}}(\mathcal{H})$ and

$$\frac{1}{2}\|\hat{\rho}_\psi^{\rm noisy} - \hat{\rho}_\psi^s\|_1 \leq \epsilon. \tag{100}$$

Thus, as a state preparation channel $\mathcal{O}[\hat{\rho}] : 1 \mapsto \hat{\rho}$, it should satisfy

$$\frac{1}{2}\|\mathcal{O}[\hat{\rho}_\psi^{\rm noisy}] - \mathcal{O}[\hat{\rho}_\psi^s]\|_\diamond \leq \epsilon. \tag{101}$$

Definition 9 ((E, s, ϵ)-independent Markovian noise model for gate)**.** Given a fixed, positive, monotonically increasing, locally bounded function $g_{\rm sup}$, consider a collection $\mathcal{C}'$ of physical systems that comprise a CV quantum computer, and regard other physical systems as environments. A noisy physical gate operation for a target physical gate $\mathcal{U}(\hat{\rho}) = \hat{U}\hat{\rho}\hat{U}^\dagger$ is said to obey an (E, s, ϵ)-independent Markovian noise model if it implements a CPTP map $\mathcal{U}^{\rm noisy} : \mathfrak{S}_E(\mathcal{H}_Q) \to \mathfrak{S}_{g_{\rm sup}(E)}(\mathcal{H}_Q)$, independent of other physical systems in $\mathcal{C}'$, such that there exists a CPTP map $\mathcal{N}^s$ defined in Def. 7 that satisfies $\mathcal{N}^s \circ \mathcal{U}(\hat{\rho}) \in \mathfrak{S}_{g_{\rm sup}(E)}(\mathcal{H}_Q)$ for any $\hat{\rho} \in \mathfrak{S}_E(\mathcal{H}_Q)$ and

$$\frac{1}{2}\left\|\mathcal{U}^{\rm noisy} - \mathcal{N}^s \circ \mathcal{U}\right\|_\diamond^E \leq \epsilon. \tag{102}$$

Definition 10 ((E, s, ϵ)-independent Markovian noise model for measurement)**.** Consider a collection $\mathcal{C}'$ of physical systems that comprise a CV quantum computer, and regard other physical systems as environments. A noisy measurement for a target measurement channel $\mathcal{M} : \mathfrak{S}_E(\mathcal{H}_Q) \to \mathcal{P}$, where $\mathcal{P}$ denotes the set of probability distribution over a binary outcome $\{0, 1\}$, is said to obey an (E, s, ϵ)-independent Markovian noise model if it implements a CPTP map $\mathcal{M}^{\rm noisy}$, independent of other physical systems in $\mathcal{C}'$, such that there exists a CPTP map $\mathcal{N}^s$ defined in Def. 7 that satisfies

$$\frac{1}{2}\|\mathcal{M}^{\rm noisy} - \mathcal{M} \circ \mathcal{N}^s\|_\diamond^E \leq \epsilon. \tag{103}$$

The meaning of a positive constant $E_{\rm prep}$ and a fixed, positive, monotonically increasing, locally bounded function $g_{\rm sup}$ in an FT-GKP circuit will be clarified in the next section. One may consider that the above definitions of the noise strength through Eqs. (100), (102), and (103) are not conventional. In fact, if we assume that our target state, gate, and measurement are $\hat{\rho}_\psi^s$, $\mathcal{N}^s \circ \mathcal{U}$, and $\mathcal{M} \circ \mathcal{N}^s$ rather than $|\overline{\psi}\rangle\langle\overline{\psi}|$, $\mathcal{U}$, and $\mathcal{M}$, respectively, then the definition reduces to a more conventional Markovian-type noise model with the noise strength measured by the energy-constrained diamond-norm distance. However, for later use, we add a parameter s in the definition here. The reader can always reproduce a more conventional picture by assuming that $\hat{\rho}_\psi^s$, $\mathcal{N}^s \circ \mathcal{U}$, and $\mathcal{M} \circ \mathcal{N}^s$ are the target state, gate, and measurement.

In the following, we give examples of (s, ϵ)-independent and (E, s, ϵ)-independent Markovian noise models that frequently appear in quantum optical experiments and in theoretical analyses of optical quantum computation. For state preparation, we analyze how the conventional approximate GKP codes studied in Ref. [15] can be interpreted as (s, ϵ)-independent Markovian noise. We consider the following standard form of an approximate GKP codeword studied in Ref. [15], which is symmetric under the Fourier transform and parameterized by only one parameter σ^2:

$$|\overline{0}_{\sigma^2}^{\rm app}\rangle := \frac{1}{\sqrt{c\sigma^2 N_{\sigma^2}}} \sum_{m\in\mathbb{Z}} e^{-4c^2\sigma^2 m^2} \int dt\, e^{-\frac{t^2}{4\sigma^2}} \,|t + 2cm\sqrt{1-4\sigma^4}\rangle_q\,, \tag{104}$$

where the normalization factor N_{σ^2} is explicitly given in Ref. [15]. The ratio in decibel between the variance of each peak of the position probability distribution of $|\overline{0}_{\sigma^2}^{\rm app}\rangle$ and that of the vacuum, i.e., $-10\log(2\sigma^2)$, is called the squeezing parameter of this approximate GKP state. From Eq. (42), we obtain the Zak-basis representation of $|\overline{0}_{\sigma^2}^{\rm app}\rangle$ state as

$$|\overline{0}_{\sigma^2}^{\rm app}\rangle = \frac{e^{iz_1z_2/2}}{\sqrt{c^2\sigma^2 N_{\sigma^2}}}\Theta\begin{bmatrix}(z_1/2c\ \ 0)^\top \\ (-z_2/c\ \ 0)^\top\end{bmatrix}(\mathbf{0}, i\Omega)\,|z_1, z_2\rangle_Z\,, \tag{105}$$

where $\Theta \begin{bmatrix} \vec{a} \\ \vec{b} \end{bmatrix} (\vec{z}, \boldsymbol{\tau}) := \sum_{\vec{s}\in\mathbb{Z}^n} \exp[\pi i(\vec{s}+\vec{a})^\top \boldsymbol{\tau}(\vec{s}+\vec{a}) + 2\pi i(\vec{z}+\vec{b})^\top(\vec{s}+\vec{a})]$ denotes the Riemann theta function [15], and the 2×2 matrix Ω is given by

$$\Omega := \frac{1}{\sigma^2}\begin{pmatrix} 1 & -\sqrt{1-4\sigma^4} \\ -\sqrt{1-4\sigma^4} & 1 \end{pmatrix}. \tag{106}$$

This state has a support all over the region $(z_1, z_2) \in [-\frac{c}{2}, \frac{3c}{2}) \times [-\frac{c}{2}, \frac{c}{2})$, and thus has a non-zero trace distance from an s-parameterized GKP state in Def. 1 for any $s < c/2$. Thus, the preparation of this type of approximate GKP state $|\overline{0}^{\mathrm{app}}_{\sigma^2}\rangle$ can be regarded as an example of an (s,ϵ)-independent Markovian noise for preparation. We thus expect that $\hat{\Pi}_s |\overline{0}^{\mathrm{app}}_{\sigma^2}\rangle$ (properly normalized) would be a valid s-parameterized GKP state and $(1-\langle\overline{0}^{\mathrm{app}}_{\sigma^2}|\,\hat{\Pi}_s\,|\overline{0}^{\mathrm{app}}_{\sigma^2}\rangle)^{1/2}$ would be the noise strength ϵ of an (s,ϵ)-independent Markovian noise. Figure 5a) in Methods plots $(1-\langle\overline{0}^{\mathrm{app}}_{\sigma^2}|\,\hat{\Pi}_s\,|\overline{0}^{\mathrm{app}}_{\sigma^2}\rangle)^{1/2}$ varying the parameter σ^2 of a conventional approximate GKP state $|\overline{0}^{\mathrm{app}}_{\sigma^2}\rangle$ in Eq. (104) against a fixed value of $s = c/14$. As the figure suggests, a conventional approximate GKP state with 25–30 dB squeezing would be almost exactly an s-parameterized GKP state for $s = c/14$. Unfortunately, the state $\hat{\Pi}_s |\overline{0}^{\mathrm{app}}_{\sigma^2}\rangle$ (after normalization) has infinite energy since a state whose wave function has a discontinuity has infinite energy in general. This causes a problem in later analysis, and we need to consider a "smoothed version" of $\hat{\Pi}_s |\overline{0}^{\mathrm{app}}_{\sigma^2}\rangle$ that has a finite energy E_{prep}. The trace distance to such a smoothed version of $\hat{\Pi}_s |\overline{0}^{\mathrm{app}}_{\sigma^2}\rangle$ may be larger than the one shown in Fig. 5a), and there may thus be a tradeoff between E_{prep} and ϵ. These will be discussed in Supplementary Note 2 C.

For the CV gate operations we listed in Supplementary Note 1 B, a bound on the energy-constrained diamond-norm distance between an ideal CV gate and its conventional experimental approximation has been studied in detail [28]. The results in Ref. [28] thus have direct interpretations as $(E, 0, \epsilon)$-independent Markovian noise. Importantly, when the identity gate (simply waiting or employing a delay line) is subject to independent photon loss on each mode (which is a dominant noise source in an optical CV system), the energy-constrained diamond-norm distance between the identity operation Id and its noisy version, i.e., the loss channel $\mathcal{L}^\eta$ with the transmissivity (transmission probability) η is given for an integer E by [28, 29]

$$\frac{1}{2}\|\mathrm{Id} - \mathcal{L}^\eta\|^E_\diamond \le \sqrt{1-\eta^E}, \tag{107}$$

and thus it can be interpreted as $(E, 0, \sqrt{1-\eta^E})$-independent Markovian noise. The nonideality caused by the conventional experimental approximations of displacement and SUM gates can also be reduced to the distance between the identity and a loss channel given above [28]. Another ubiquitous noise in an optical CV system is a random phase rotation. The energy-constrained diamond-norm distance between a random phase-rotation channel and the identity channel can be computed as [17]

$$\frac{1}{2}\left\|\mathrm{Id} - \int_{-\pi}^{\pi} d\theta\, f(\theta)\mathcal{R}[\theta]\right\|^E_\diamond \le \int_{-\pi}^{\pi} d\theta\, f(\theta)\sqrt[3]{4|\theta|E}, \tag{108}$$

where $f(\theta)$ is a probability distribution over $[-\pi,\pi)$, typically well peaked at $\theta = 0$. Figures 5c) and d) in Methods show estimates of the parameter ϵ when $f(\theta)$ is a wrapped normal distribution with the variance v, i.e.,

$$f(\theta) = \frac{1}{2\pi}\,\Theta\begin{bmatrix}0\\0\end{bmatrix}\left(0, \frac{iv}{2\pi}\right). \tag{109}$$

Figure 5c) plots the value ϵ for a fixed value of $v = 5\times 10^{-5}$ as varying the energy constraint E. Figure 5d), on the other hand, plots the value ϵ for a fixed input energy $E = 20$ as varying the variance v of the wrapped normal distribution. These plots show the required level of accuracy of the phase control in CV experiments to achieve the fault tolerance.

From the definition of (E,s,ϵ)-independent Markovian noise in Def. 9, Eqs. (107) and (108) lead to (E,s,ϵ)-independent Markovian noise with $s \ge 0$ as well. There may, however, be a tradeoff between s and ϵ in general, meaning that the above evaluations of ϵ independent of s are not tight in general. Tighter evaluation of ϵ depending on s may be possible by using the characteristic function formula for these channels given in Ref. [1], but we leave it for future work.

On the other hand, we have an s-dependent upper bound on ϵ for a random-displacement channel $\mathcal{V}[p]$ regarded as an (E,s,ϵ)-independent Markovian noise, where $\mathcal{V}[p]$ is defined as

$$\mathcal{V}[p](\hat{\rho}) := \iint_{\mathbb{R}} dz_1 dz_2\; p(z_1,z_2)\hat{V}(z_1,z_2)\hat{\rho}\hat{V}(z_1,z_2)^\dagger. \tag{110}$$

Let $\widetilde{\mathcal{V}}^s[p]$ be an s-parameterized noise channel defined as

$$\widetilde{\mathcal{V}}^s[p](\hat{\rho}) := \left(1 - \iint_{-s}^{s} dz_1 dz_2\, p(z_1, z_2)\right)\hat{\rho} + \iint_{-s}^{s} dz_1 dz_2\, p(z_1, z_2)\hat{V}(z_1, z_2)\hat{\rho}\hat{V}(z_1, z_2)^\dagger. \tag{111}$$

Then, we have

$$\frac{1}{2}\|\mathcal{V}[p] - \widetilde{\mathcal{V}}^s[p]\|_\diamond^E$$

$$\leq \frac{1}{2}\left(1 - \iint_{-s}^{s} dz_1 dz_2\, p(z_1, z_2) + \sup_{\hat{\rho}_{RQ}:\hat{\rho}_Q \in \mathfrak{S}_E(\mathcal{H}_Q)} \left\| \int_{|z_1|>s} \int_{|z_2|>s} dz_1 dz_2\, p(z_1, z_2)\hat{V}_Q(z_1, z_2)\hat{\rho}_{RQ}\hat{V}_Q(z_1, z_2)^\dagger \right\|\right) \tag{112}$$

$$\leq 1 - \iint_{-s}^{s} dz_1 dz_2\, p(z_1, z_2), \tag{113}$$

which should be small as long as the probability distribution p is well peaked at the origin. Note that this bound is independent of E, as opposed to the previous examples (107) and (108). Figure 5b) in Methods plots the parameter ϵ of (E, s, ϵ)-independent Markovian noise when $s = c/182$ and $p(z_1, z_2)$ is a Gaussian function with the variance V in both quadratures, i.e.,

$$p(z_1, z_2) = \frac{\exp[-(z_1^2 + z_2^2)/(2V)]}{2\pi V}. \tag{114}$$

For measurement, experimental homodyne detectors generally have a finite resolution b; i.e., the measurement outcome is discretized with a bin size b. It also has a finite range Γ; i.e., the value of $\hat{q}$ larger (resp. smaller) than Γ (resp. $-\Gamma$) is rounded to Γ (resp. $-\Gamma$). A binned homodyne detection for $\hat{q}$ with a bin size b can be interpreted as the ideal homodyne detection followed by the binning with the size b, but then, this is equivalent to $\{\hat{q}\}_b$-dependent displacement before the ideal homodyne detection. Since it is up-to-b displacement noise in the $\hat{q}$ quadrature, it can be regarded as an $(E, b, 0)$-independent Markovian noise for any E. On the other hand, the effect of a finite range Γ should depend on the energy E of an input state since a state with large E has more probability to be observed in $|\hat{q}| \geq \Gamma$. Using the fact that $\langle \hat{q}^2 \rangle_{\hat{\rho}} \leq 2\langle \hat{n} \rangle_{\hat{\rho}} + 1 \leq 2E + 1$ for any $\hat{\rho} \in \mathfrak{S}_E(\mathcal{H})$, we have

$$\Pr\left[|\hat{q}| > \Gamma \mid \hat{\rho} \in \mathfrak{S}_E(\mathcal{H})\right] = \Pr\left[\hat{q}^2 > \Gamma^2 \mid \hat{\rho} \in \mathfrak{S}_E(\mathcal{H})\right] \leq \frac{\langle \hat{q}^2 \rangle_{\hat{\rho}}}{\Gamma^2} \leq \frac{2E + 1}{\Gamma^2}, \tag{115}$$

where we used Markov's inequality in the first inequality. Thus, for an experimental approximation $\mathcal{M}_{\mathrm{hom}}^{\mathrm{noisy}}[\Gamma]$ with the range Γ of the ideal homodyne detector $\mathcal{M}_{\mathrm{hom}}^{\mathrm{ideal}}$ satisfies

$$\frac{1}{2}\|\mathcal{M}_{\mathrm{hom}}^{\mathrm{ideal}} - \mathcal{M}_{\mathrm{hom}}^{\mathrm{noisy}}[\Gamma]\|_\diamond^E \leq \frac{2E + 1}{\Gamma^2}, \tag{116}$$

and can thus be regarded as an $(E, 0, (2E + 1)/\Gamma^2)$-independent Markovian noise model for measurement. Since Eq. (116) still holds even if we perform post-processing after both $\mathcal{M}_{\mathrm{hom}}^{\mathrm{ideal}}$ and $\mathcal{M}_{\mathrm{hom}}^{\mathrm{noisy}}[\Gamma]$, an experimentally realizable homodyne detector with the resolution b and the range Γ can be regarded as an $(E, b, (2E + 1)/\Gamma^2)$-independent Markovian noise.

Supplementary Note 2. GENERAL FAULT TOLERANCE FOR THE GKP CODE

A. Construction of GKP gadgets and a fault-tolerant GKP circuit

In a fault-tolerant circuit, each location C_i of an original circuit C is replaced with a "gadget" to implement such operations in a fault-tolerant manner [13]. In a concatenated code, for example, each location of the circuit C in the nth level of concatenation consists of a circuit with the $(n-1)^{\mathrm{th}}$-level code and fault-tolerant operations with it. Since the GKP code encodes a qubit in a CV system, each location in the original qubit circuit C is, in this case, replaced with physical CV operations listed in Supplementary Note 1 B. Explicitly, we define a fault-tolerant GKP gadget for each qubit circuit element as follows.

Preparation:

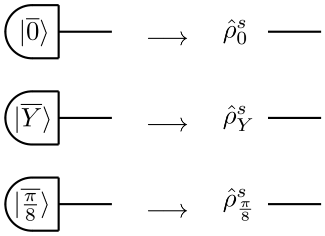

(117)

(118)

(119)

Gate:

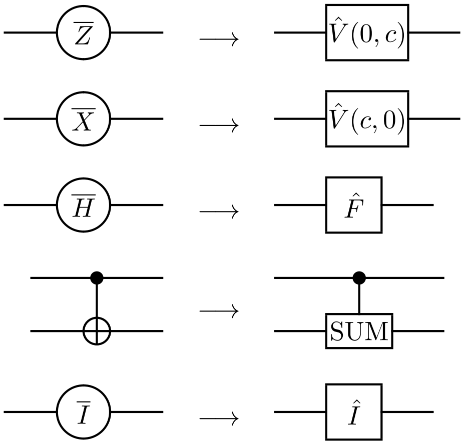

(120)

(121)

(122)

(123)

(124)

Measurement:

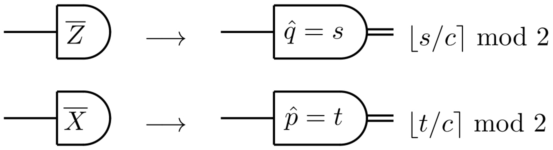

(125)

(126)

In the above, $\lfloor a \rceil$ denotes the rounding of a defined in Eq. (17). In the following, we may omit writing the wait operations (identity gates) explicitly in the circuits unless necessary. With these primitive gadgets, we can construct a universal gate set in Eq. (49). The logical phase gate $\overline{S}$ can be realized by the following catalytic circuit [30].

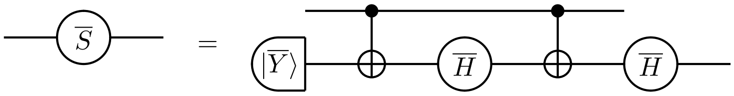

(127)

On the right-hand side above, the phase gate applies to the first qubit. The last $\overline{H}$ gate on the second wire resets a state to $|\overline{Y}\rangle$, which can replace a $|\overline{Y}\rangle$-state preparation for later $\overline{S}$ gates. Conventionally, the phase gate for the GKP code is realized by the shear $\exp(i\hat{q}^2/2)$, but to simplify a fault-tolerance condition stated in this section, we assume the $|\overline{Y}\rangle$-state preparation and the above catalytic circuit instead. As mentioned later, we can use the conventional gate $\exp(i\hat{q}^2/2)$ instead of $|\overline{Y}\rangle$ preparation at the cost of a slight modification of the fault-tolerance condition. Since all the Clifford gates are generated by the phase gate, the Hadamard gate $\overline{H}$, and the CNOT gate, we conclude that the above gadgets are sufficient to realize the Clifford operations. Furthermore, when we combine the $|\frac{\pi}{8}\rangle$ state with the Clifford gates, we can realize the $\overline{T}$ gate through gate teleportation as follows [4].

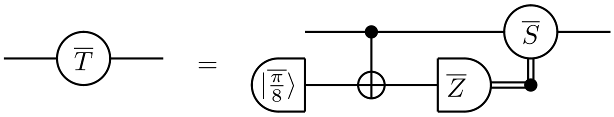

(128)

The gadgets in Eqs. (117)–(128) implement the universal gate set in Eq. (49), as well as the computational-basis state preparation and measurement.

For the theory of fault tolerance, we also need to construct a GKP error-correction (EC) gadget. There are several options for this, but here we use the Knill-type EC gadget [16, 31] for several reasons, which will be made clear later.

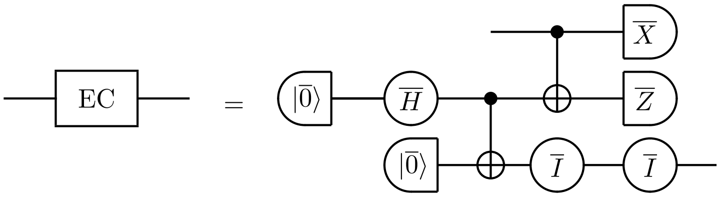
(129)

In the above, we omit the correction operation. The correction operation can instead be taken into account by updating the Pauli frame [32] or by changing a successive gate or measurement.

Using these GKP gadgets, we make a precise definition of our fault-tolerant protocol. Our protocol is constructed based on the protocol for concatenated codes in Ref. [13], but we only aim at describing how a qubit-level circuit can be constructed by a fault-tolerant CV circuit and how a noise property on this qubit-level circuit is determined by the underlying CV circuit. Thus, the qubit-level circuit may also need to be encoded by a concatenated code as in Ref. [13]. Putting this in mind, we define the fault-tolerant-GKP (FT-GKP) circuit as follows.

Definition 11 (FT-GKP circuit)**.** Let C be a circuit on qubits. Let $\{C_i\}_{i\in\mathcal{I}}$ be a set of locations of C, where each C_i is a preparation location, a measurement location, a gate location, or a wait location. We assume that the circuit C can be divided so that every qubit is involved in exactly one location at each time step. Two locations are considered *consecutive* if they occur at adjacent time steps and share a qubit. Given C, the *fault-tolerant-GKP (FT-GKP) protocol* provides a CV circuit C' constructed by replacing each location C_i with a GKP gadget C'_i for C_i and adding GKP EC gadgets between any pair of consecutive locations. The circuit C' is referred to as an *FT-GKP circuit* for C.

As stated previously, the above definition is not a whole protocol for fault tolerance; the qubit circuit C should be a qubit version of an FT implementation of an original circuit C_{orig} representing computation in the sense of Ref. [13]. If the concatenation level of the qubit circuit C is enough to correct logical errors of the FT-GKP circuit, the whole protocol will be fault-tolerant. Our analysis in the following will translate a noise model in CV systems into a noise model at a qubit level so that we can use the established fault-tolerant protocols at a qubit level to achieve fault tolerance.

B. Fault-tolerance conditions for GKP gadgets

In Ref. [13], they consider the equivalence class of faulty gadgets in which at most s locations inside a gadget are faulty. Then, the fault-tolerance condition requires that a gadget with s faulty locations causes at most weight-s errors, i.e., errors act on s qubits that comprise a code. If only this condition is satisfied, the whole protocol can be shown to be fault-tolerant for any errors that occur according to an appropriate noise model. Although these definitions have a clear meaning in qubit concatenated error-correcting codes, they do not in the GKP code. After all, CV systems should be in the bottom layer of concatenation in our protocol, so the faults at the physical level are directly caused by the physical noise of CV systems rather than qubits. We need to find an alternative equivalence class of faults or errors and the corresponding fault-tolerance criteria in the CV case.

The GKP code is introduced to correct displacement errors in the phase space up to $c/2$ with c in Eq. (6). Thus, a natural parameterization of errors should be the "amount" of displacement. To make this statement rigorous, we introduce the following family of projection operators, which is an appropriate modification of the filter operator in Ref. [13] for the GKP code.

Definition 12 (The stabilizer-subsystem (SSS) r-filter and the ideal GKP decoder)**.** Let $0 < r \leq c/2$. The SSS r-filter $\hat{\Pi}_r$ is defined as

$$\hat{\Pi}_r := \sum_{\mu=0,1}\int_{|z_1|<r} dz_1 \int_{|z_2|<r} dz_2 \; |\mu; z_1, z_2\rangle\langle\mu; z_1, z_2|_{LS} = \hat{I}_L \otimes \int_{|z_1|<r} dz_1 \int_{|z_2|<r} dz_2 \; |z_1, z_2\rangle\langle z_1, z_2|_S \,. \tag{130}$$

The ideal GKP decoder $\mathcal{D}_{\mathrm{GKP}}$ is a map from a CV system to a qubit, defined as

$$\mathcal{D}_{\mathrm{GKP}}(\hat{\rho}) := \sum_{\mu,\nu\in\{0,1\}}\int_{|z_1|<\frac{c}{2}} dz_1 \int_{|z_2|<\frac{c}{2}} dz_2 \; |\mu\rangle\langle\mu; z_1, z_2|_{LS}\, \hat{\rho}\, |\nu; z_1, z_2\rangle_{LS}\langle\nu| = \mathrm{Tr}_S(\hat{\rho}). \tag{131}$$

The SSS r-filter is a tool to know how much a state is effectively displaced away from the "ideal GKP code space", and the ideal GKP decoder is a tool to know a reduced state on the logical qubit of the SSS decomposition. One can check from Eqs. (27) and (28) that the SSS r-filter is a projection operator and satisfies $\hat{\Pi}_{\frac{c}{2}} = \hat{I}$. One should be careful, however, that $\lim_{r\to 0} \hat{\Pi}_r$ does not converge to a projection onto the "ideal GKP code space" but the zero operator in the strong operator topology. The SSS r-filter and the ideal GKP decoder will be represented graphically as follows.

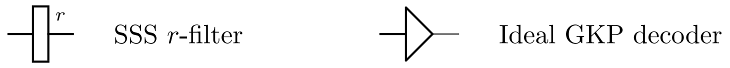

Now, we define a faulty version of the gadgets introduced in the previous section, which are again generalizations of Ref. [13] for our scenario. We start with the definition of an s-parameterized GKP preparation. This definition is the same as the one defined in the previous section, but we explicitly show the dependence on the parameter s in Def. 1. Alternatively, one can consider an s-preparation as an ideal s_1-parameterized GKP state preparation followed by a noise map $\mathcal{N}^{s_2}$ defined in Def. 7, but this is again an (s_1+s_2)-parameterized GKP state preparation as can be seen from Eq. (51).

Definition 13 (s-preparation)**.** An s-parameterized GKP preparation (s-preparation for short) gadget is defined as follows.

s-preparation: 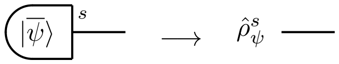 (132)

In the above, ψ denotes either 0, Y, or $\frac{\pi}{8}$, and $\hat{\rho}^s_\psi$ is as defined in Def. 1.

For an s-parameterized GKP gate gadget, the parameter s denotes the degree of displacement noise added during these operations. When noise is Markovian, the right-hand side in the following definition includes general noise models as $s\to\infty$, since any CPTP map $\mathcal{N}_U$ that is a noisy version of a gate $\hat{U}$ can be decomposed as $(\mathcal{N}_U \circ \mathcal{U}^{-1}) \circ \mathcal{U}$, where $\mathcal{U}(\hat{\rho}) = \hat{U}\hat{\rho}\hat{U}^{-1}$, and the channel $\mathcal{N}_U \circ \mathcal{U}^{-1}$ can be expanded by displacement operators.

Definition 14 (s-gate)**.** Let us define an s-parameterized GKP gate gadget (s-gate for short) as follows.

s-gate: 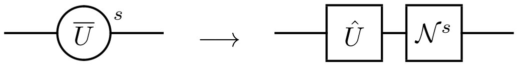 (133)

In the above, $\hat{U}$ is either $\hat{V}(0,c)$, $\hat{V}(c,0)$, $\hat{F}$, $\exp(-i\hat{q}_1\hat{p}_2)$, or $\hat{I}$, and $\mathcal{N}^s$ is defined in Def. 7.

In the same way, we define an s-parameterized measurement gadget below. As opposed to an s-gate case, the right-hand side of the definition below may not be a general noise model for measurement even when noise is Markovian and $s\to\infty$ since general POVM may not necessarily be represented as a noise channel followed by an ideal homodyne detection and the GKP binning. More general POVMs can instead be approximated by the POVM of the form in Eq. (134) in the energy-constrained diamond-norm distance as considered in Eq. (103).

Definition 15 (s-measurement)**.** An s-parameterized GKP measurement gadget (s-measurement for short) is defined as follows.

s-measurement: 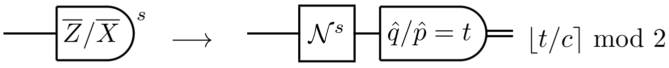 (134)

Finally, we need to introduce a parameterized version of the EC gadget. An s-EC gadget should reset the degree of displacement. This resetting of a wave function in the GKP error correction is first pointed out in Ref. [33] for Gaussian random displacement noise, but here we make the statement more rigorous and applicable even beyond Gaussian noise using the tools we developed so far.

Definition 16 (s-EC)**.** We define an s-parameterized EC gadget as follows, replacing all the circuit elements in Eq. (129) with s-preparations, s-gates, and s-measurements:

s-EC: 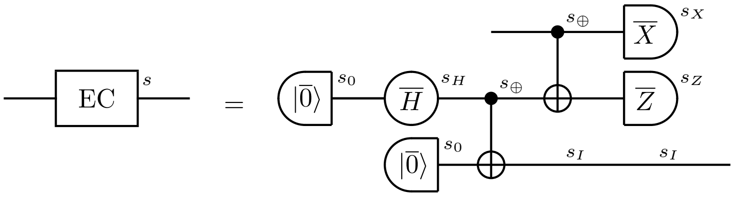 , (135)

where the parameter s is given as a function of s_0, s_H, $s_\oplus$, s_X, and s_Z as

$$s := 2s_0 + s_H + s_\oplus + \max\{s_\oplus + \max\{s_X, s_Z\}, 2s_I\}. \tag{136}$$

Having defined parameterized gadgets, we can state a fault-tolerance (FT) condition for these gadgets, which will be used later. The following proposition shows that an s-measurement works as if an incoming mode is decoded by the ideal GKP decoder followed by the logical qubit measurement as long as a total displacement by $r+s$ is below $\frac{c}{2}$. As the proof suggests, it is the homodyne detection that allows an s-measurement gadget to satisfy the FT condition. However, other implementations of a measurement gadget are not prohibited in principle as long as the above condition is satisfied.

Proposition 17 (FT condition for s-measurement)**.** A GKP s-measurement gadget defined in Def. 15 satisfies the following:

Meas: 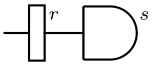

= 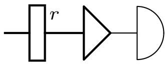

when $r+s<\frac{c}{2}$, where the thin right half circle denotes a qubit measurement.

Proof. We consider the case of $\overline{Z}$ measurement, while the same argument holds for $\overline{X}$ measurement. From Eq. (47), the POVM elements of the ideal GKP $\overline{Z}$ measurement can be given by

$$\left\{|0\rangle\langle 0|_L \otimes \hat{I}_S, |1\rangle\langle 1|_L \otimes \hat{I}_S\right\} = \left\{\int_{-\frac{c}{2}}^{\frac{c}{2}} dz_1 \int_{-\frac{c}{2}}^{\frac{c}{2}} dz_2\, |0; z_1, z_2\rangle\langle 0; z_1, z_2|_{LS}\, , \int_{-\frac{c}{2}}^{\frac{c}{2}} dz_1 \int_{-\frac{c}{2}}^{\frac{c}{2}} dz_2\, |1; z_1, z_2\rangle\langle 1; z_1, z_2|_Z\right\}. \tag{137}$$

Therefore, for any density operator $\hat{\rho}$ and for any noise channel $\mathcal{N}^s$ whose Kraus operators are linear combinations of elements in $\{\hat{V}(z_1,z_2) : (z_1,z_2) \in [-s,s) \times [-s,s)\}$, we have, for $\mu \in \{0,1\}$,

$$\begin{aligned}
&\mathrm{Tr}\left[(|\mu\rangle\langle\mu|_L \otimes \hat{I}_S)\mathcal{N}^s(\hat{\Pi}_r\hat{\rho}\hat{\Pi}_r)\right] \\
&= \mathrm{Tr}\left[(|\mu\rangle\langle\mu|_L \otimes \hat{I}_S)\, \mathcal{N}^s\left(\sum_{\nu=0,1}\iint_{|z_1|,|z_2|<r} dz_1 dz_2\, |\nu; z_1, z_2\rangle\langle \nu; z_1, z_2|\, \hat{\rho} \sum_{\nu'=0,1}\iint_{|z_1'|,|z_2'|<r} dz_1' dz_2'\, |\nu'; z_1', z_2'\rangle\langle \nu'; z_1', z_2'|\right)\right] \qquad (138) \\
&= \mathrm{Tr}\Bigg[(|\mu\rangle\langle\mu|_L \otimes \hat{I}_S) \int_{\mathbb{R}^4} d^2w d^2w'\, \mathcal{E}_s(w_1, w_2, w_1', w_2') \\
&\qquad \sum_{\nu,\nu'=0,1}\iiiint_{|z_1|,|z_2|,|z_1'|,|z_2'|<r} dz_1 dz_2 dz_1' dz_2'\, \hat{V}(w_1,w_2)\, |\nu; z_1, z_2\rangle\langle \nu; z_1, z_2|\, \hat{\rho}\, |\nu'; z_1', z_2'\rangle\langle \nu'; z_1', z_2'|\, \hat{V}(w_1', w_2')^\dagger\Bigg] \qquad (139) \\
&= \mathrm{Tr}\Bigg[(|\mu\rangle\langle\mu|_L \otimes \hat{I}_S) \int_{\mathbb{R}^4} d^2w d^2w'\, \mathcal{E}_s(w_1, w_2, w_1', w_2') e^{i(w_2 z_1 - w_1 z_2)/2 - i(w_2' z_1' - w_1' z_2')/2} \\
&\qquad \sum_{\nu,\nu'=0,1}\iiiint_{|z_1|,|z_2|,|z_1'|,|z_2'|<r} dz_1 dz_2 dz_1' dz_2'\, |\nu; z_1 + w_1, z_2 + w_2\rangle\langle \nu; z_1, z_2|\, \hat{\rho}\, |\nu'; z_1', z_2'\rangle\langle \nu'; z_1' + w_1', z_2' + w_2'|\Bigg], \qquad (140)
\end{aligned}$$

where we used Eq. (99) in the second equality. Recall that $\mathcal{E}_s(z_1', z_2', z_1'', z_2'')$ has a support only in $\prod_{i=1}^4 [-s,s)$, and thus the right-hand side of (140) has nonzero values only for $|z_i + w_i| \le r + s < \frac{c}{2}$ and $|z_i' + w_i'| \le r+s < \frac{c}{2}$. Therefore, we have

$$\begin{aligned}
(140) = \mathrm{Tr}\Bigg[&\int_{\mathbb{R}^4} d^2w d^2w'\, \mathcal{E}_s(w_1, w_2, w_1', w_2') e^{i(w_2 z_1 - w_1 z_2)/2 - i(w_2' z_1' - w_1' z_2')/2} \\
&\iiiint_{|z_1|,|z_2|,|z_1'|,|z_2'|<r} dz_1 dz_2 dz_1' dz_2'\, |\mu; z_1 + w_1, z_2 + w_2\rangle\langle \mu; z_1, z_2|\, \hat{\rho}\, |\mu; z_1', z_2'\rangle\langle \mu; z_1' + w_1', z_2' + w_2'|\Bigg]
\end{aligned} \tag{141}$$

$$= \mathrm{Tr}\Bigg[\int_{\mathbb{R}^4} d^2w d^2w' \; \mathcal{E}_s(w_1, w_2, w_1', w_2')$$
$$\iiiint_{|z_1|,|z_2|,|z_1'|,|z_2'|<r} dz_1 dz_2 dz_1' dz_2' \; \hat{V}(w_1, w_2) \, |\mu; z_1, z_2\rangle\langle\mu; z_1, z_2| \, \hat{\rho} \, |\mu; z_1', z_2'\rangle\langle\mu; z_1', z_2'| \, \hat{V}(w_1', w_2')^\dagger \Bigg] \tag{142}$$

$$= \mathrm{Tr}\Bigg[\mathcal{N}^s \left(\iiiint_{|z_1|,|z_2|,|z_1'|,|z_2'|<r} dz_1 dz_2 dz_1' dz_2' \;\; |\mu; z_1, z_2\rangle\langle\mu; z_1, z_2| \, \hat{\rho} \, |\mu; z_1', z_2'\rangle\langle\mu; z_1', z_2'| \right)\Bigg] \tag{143}$$

$$= \mathrm{Tr}[\langle\mu|_L \, \hat{\Pi}_r \hat{\rho} \hat{\Pi}_r \, |\mu\rangle_L] \tag{144}$$

$$= \langle\mu|_L \, \mathrm{Tr}_S[\hat{\Pi}_r \hat{\rho} \hat{\Pi}_r] \, |\mu\rangle_L \, , \tag{145}$$

where we used the fact that $\mathcal{N}^s$ is trace-preserving in the second last equality. This proves the statement. Q.E.D.

Next, we state the FT condition for a preparation gadget.

Proposition 18 (FT condition for s-preparation)**.** A GKP s-preparation gadget defined in Def. 13 satisfies the following conditions.

Prep A: 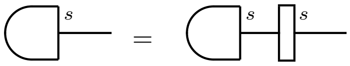 when $s < \frac{c}{2}$.

Prep B: 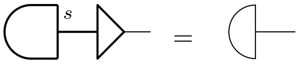 when $s < \frac{c}{2}$, where the thin left half circle denotes a qubit state preparation.

Proof. It suffices to show that the mentioned property holds for any i in the mixture of Eq. (51). When $s < \frac{c}{2}$, an s-parameterized state defined in Eq. (52) can be rewritten as

$$|\rho_i^s[\psi]\rangle = |\psi\rangle_L \otimes \int_{-\frac{c}{2}}^{\frac{c}{2}} dz_1 \int_{-\frac{c}{2}}^{\frac{c}{2}} dz_2 \; f_s^i(z_1, z_2) \, |z_1, z_2\rangle_S \, . \tag{146}$$

From this and the definition of the SSS r-filter in Eq. (130), we have $\hat{\Pi}_s \, |\rho_i^s[\psi]\rangle = |\rho_i^s[\psi]\rangle$ when $s < \frac{c}{2}$, which proves Prep A. Prep B also follows from the same argument. Q.E.D.

We then state the FT condition for an s-gate, which is done similarly to the previous gadgets.

Proposition 19 (FT condition for s-gate)**.** A GKP s-gate gadget defined in Def. 14 satisfies the following conditions:

Gate A: 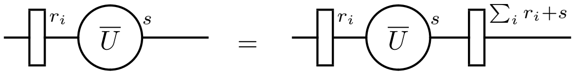 when $\sum_i r_i + s < \frac{c}{2}$.

Gate B: 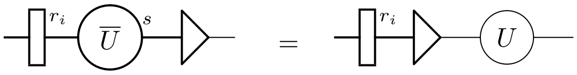 when $\sum_i r_i + s < \frac{c}{2}$, where i runs over all the modes that this gate gadget acts on, and the thin circle denotes a qubit unitary.

Proof. We only need to check this condition for our gate set $\overline{Z}$, $\overline{X}$, $\overline{H}$, and CNOT. From Eqs. (34)–(37), we have that when $r < \frac{c}{2}$,

$$\hat{V}(0, c) \hat{\Pi}_r \hat{V}(0, c)^\dagger = \hat{\Pi}_r, \tag{147}$$

$$\hat{V}(c, 0) \hat{\Pi}_r \hat{V}(c, 0)^\dagger = \hat{\Pi}_r, \tag{148}$$

$$\hat{F} \hat{\Pi}_r \hat{F}^\dagger = \hat{\Pi}_r, \tag{149}$$

where we used that the SSS r-filter is invariant under sign changes of z_1 and z_2 inside the integral. For the SUM gate, we have from Eq. (40) that when $r_j + r_k < \frac{c}{2}$,

$$\exp(-i\hat{q}_j \hat{p}_k)(\hat{\Pi}_{r_j} \otimes \hat{\Pi}_{r_k}) \exp(i\hat{q}_j \hat{p}_k) = \int_{|z_1|<r_j} dz_1 \int_{|z_2|<r_j} dz_2 \int_{|z_1'|<r_k} dz_1' \int_{|z_2'|<r_k} dz_2' \, (\hat{I}_L \otimes |z_1, z_2 - z_2'\rangle\langle z_1, z_2 - z_2'|_S)_j$$
$$\otimes (\hat{I}_L \otimes |z_1' + z_1, z_2'\rangle\langle z_1' + z_1, z_2'|_S)_k \tag{150}$$

$$\leq \int_{|z_1|<r_j} dz_1 \int_{|z_2|<r_j+r_k} dz_2 \int_{|z'_1|<r_k+r_j} dz'_1 \int_{|z'_2|<r_k} dz'_2 \, (\hat{I}_L \otimes |z_1, z_2\rangle\langle z_1, z_2|_S)_j \otimes (\hat{I}_L \otimes |z'_1, z'_2\rangle\langle z'_1, z'_2|_S)_k \tag{151}$$

$$\leq \hat{\Pi}_{r_j+r_k} \otimes \hat{\Pi}_{r_k+r_j}. \tag{152}$$

Therefore, when $r_j + r_k < \frac{c}{2}$, we have

$$\exp(-i\hat{q}_j\hat{p}_k)(\hat{\Pi}_{r_j} \otimes \hat{\Pi}_{r_k}) = \exp(-i\hat{q}_j\hat{p}_k)(\hat{\Pi}_{r_j} \otimes \hat{\Pi}_{r_k}) \exp(i\hat{q}_j\hat{p}_k) \exp(-i\hat{q}_j\hat{p}_k)(\hat{\Pi}_{r_j} \otimes \hat{\Pi}_{r_k}) \tag{153}$$

$$= \hat{\Pi}_{r_j+r_k} \otimes \hat{\Pi}_{r_k+r_j} \exp(-i\hat{q}_j\hat{p}_k)(\hat{\Pi}_{r_j} \otimes \hat{\Pi}_{r_k}), \tag{154}$$

where we used the fact that $\hat{P}\hat{Q} = \hat{Q}$ when $\hat{P} \geq \hat{Q}$ for two projection operators $\hat{P}$ and $\hat{Q}$. With these and the definition of an s-gate in Eq. (133), the condition Gate A holds for all gate gadgets as long as $\sum_j r_j + s < \frac{c}{2}$. The condition Gate B also holds from the above and Eqs. (34), (35), (36), and (40). Q.E.D.

It is the choice of the physical CV gates implementing these gadgets that allows them to satisfy the FT conditions. In other words, other choices of CV physical gates to implement gate gadgets are allowed as long as the constructed gate gadgets satisfy the above FT conditions. Here, we can explain why we do not use the conventional CV shear gate $\exp(i\hat{q}^2/2)$ to implement the GKP logical phase gate: the GKP phase gate, composed of the CV shear gate, will satisfy neither Gate A nor Gate B. To include $\exp(i\hat{q}^2/2)$ as a fault-tolerant gadget, we need to modify the FT conditions so that $\exp(i\hat{q}^2/2)$ can satisfy them. Concretely, we should modify conditions such that the last SSS r-filter $\hat{\Pi}_{r+s}$ in the right-hand side of Gate A is replaced with $\hat{\Pi}_{2r+s}$ for a single-mode gate, and both Gate A and Gate B equations only hold when $2r + s < \frac{c}{2}$ for a single-mode gate. To avoid the complication, however, we stick to our gate set introduced in Supplementary Note 1 B hereafter.

We also remark that a non-Gaussian unitary gate does not satisfy the above criteria in general. This is because non-Gaussian unitary transforms a displacement to a Gaussian unitary, which then requires infinitely many displacement operators to approximate it with a linear combination. In terms of the stabilizer subsystem decomposition, this means that even a "wave function" on the size-$\sqrt{\pi}$ Cartesian square of the syndrome subsystem that is supported in a small neighborhood of the origin might be spread across the whole region in the Cartesian square by the action of non-Gaussian unitary, which may not be correctable. Perhaps, there may exist a class of non-Gaussian gates whose effects are limited enough to preserve fault tolerance, but we leave this to future work. To avoid these complications, we implement a GKP non-Clifford gate via teleportation of a magic state [3, 34, 35] in our analysis. The FT condition for a non-Clifford gate is therefore reduced to the FT condition for a magic state preparation explained previously.

Finally, an FT condition for an s-EC gadget is given in the following.

Proposition 20 (FT condition for s-EC)**.** A GKP s-EC gadget satisfies the following conditions:

EC A: 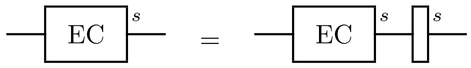 when $s < \frac{c}{2}$.

EC B: 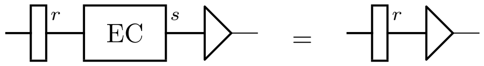 when $r + s < \frac{c}{2}$.

Proof. Due to our construction of an s-EC gadget in Eq. (135) and Proposition 17–19, we have the following chain of equalities when $2s_0 + s_H + s_\oplus + 2s_I < \frac{c}{2}$.

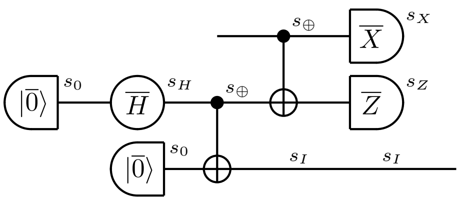 (155)

= 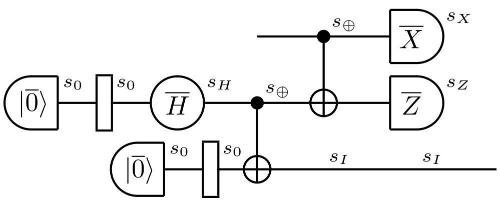 (156)

$=$ 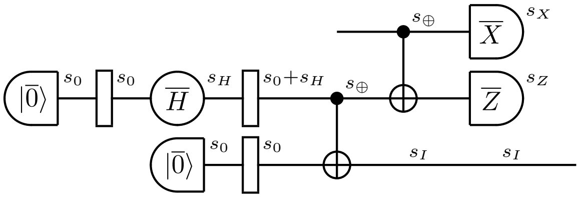 (157)

$=$ 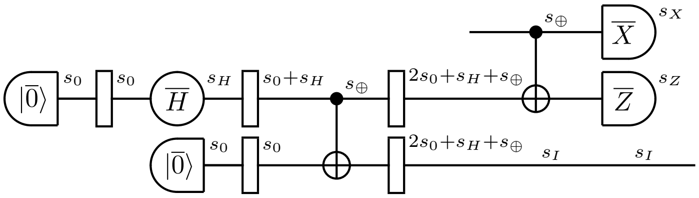 (158)

$=$ 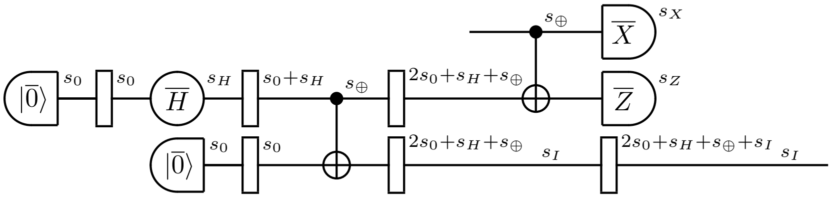 (159)

$=$ 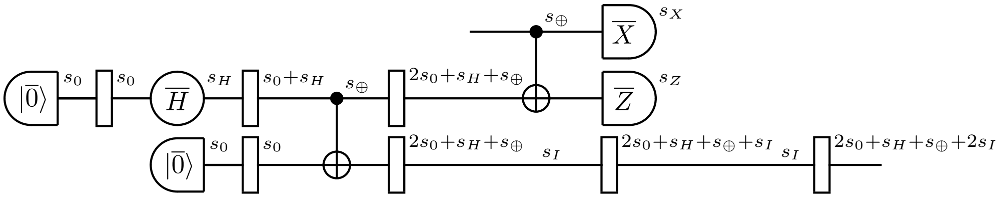 (160)

Since $2s_0 + s_H + s_\oplus + 2s_I \leq s$ always holds with s given in Eq. (136), we have the condition EC A. Furthermore, when the SSS r-filter is inserted to the input, the ideal decoder is inserted to the output, $r{+}2s_0{+}s_H{+}2s_\oplus{+}\max\{s_X, s_Z\} < \frac{c}{2}$, and $2s_0 + s_H + s_\oplus + 2s_I < \frac{c}{2}$, we have

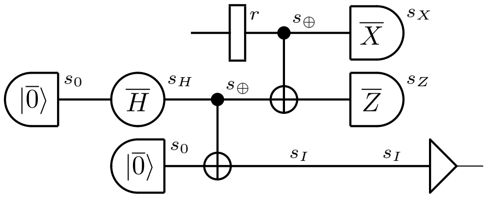 (161)

$=$ 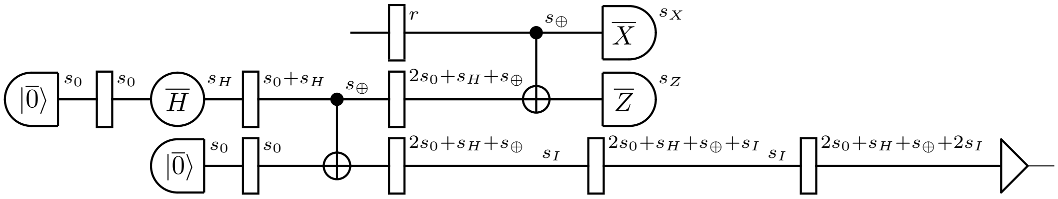 (162)

$=$ 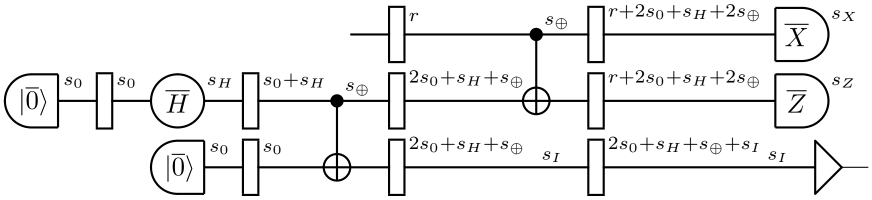 (163)

= 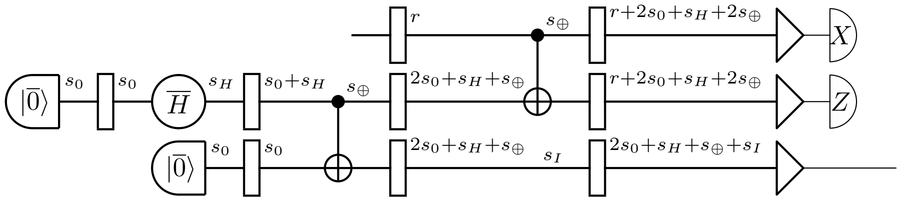 (164)

= 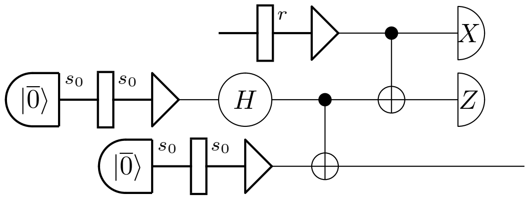 (165)

= 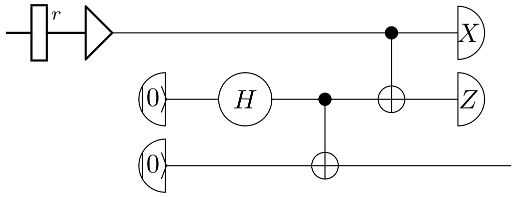 (166)

= 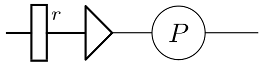 (167)

where $P \in \{I, X, Y, Z\}$ denotes a Pauli correction depending on the measurement outcomes. Due to the definition of s in Eq. (136), we have the condition EC B up to a qubit Pauli correction. As we stated previously, this correction operation can be dealt with by a change of the Pauli frame or a change of a successive gate or measurement. Q.E.D.

One thing to note is that when $r + s \geq \frac{c}{2}$, the Pauli error correction P may not be inferred correctly. This causes the qubit-level logical error, which is passed on to the higher level to be corrected by a qubit quantum error-correcting code. The detail will be described in Supplementary Note 2 D.

C. Energy-constraint conditions for GKP gadgets

As has already been discussed in Supplementary Note 1 C, a quantum state on a mode Q during computation should be an element of $\mathfrak{S}_E(\mathcal{H}_Q)$ with a finite E. This is necessary for eliminating unrealistic quantum states that have arbitrarily high susceptibility to a given noise, and thus for applying the appropriate distance measure (i.e., the energy-constrained diamond-norm distance) to noise. Thus, our noisy quantum circuit needs to maintain a quantum state during computation in $\mathfrak{S}_E(\mathcal{H}_Q)$. Furthermore, we need to compare our noisy quantum circuit with an "ideal" quantum circuit, which always gives us a correct result. Thus, the "ideal" circuit should also keep the energy of a quantum state during computation finite. Otherwise, the noise models we can treat will be severely restricted. For these reasons, we make additional requirements on noisy gadgets that one implements in an experiment as well as s-parameterized gadgets in Defs. 13 and 14.

Definition 21 (E_{prep}-energy constraint on preparation gadgets)**.** For a positive constant E_{prep}, a preparation gadget is said to satisfy the E_{prep}-energy constraint if the output state of the gadget is an element of $\mathfrak{S}_{E_{\text{prep}}}(\mathcal{H}_Q)$.

Definition 22 (g_{sup}-energy constraint on gate gadgets)**.** For a positive, monotonically increasing, locally bounded function $g_{\text{sup}}(E)$ of $E \in [0, \infty)$, a single-mode gate gadget is said to satisfy the g_{sup}-energy constraint if it outputs a quantum state that is contained in $\mathfrak{S}_{g_{\text{sup}}(E)}(\mathcal{H}_Q)$ when an element of $\mathfrak{S}_E(\mathcal{H}_Q)$ is input for any $E \in [0, \infty)$. A two-mode

gate gadget is said to satisfy the g_{sup}-energy constraint if for any input state whose reduced states on respective modes Q_1 and Q_2 are elements of $\mathfrak{S}_{g_{\mathrm{sup}}(E_1)}(\mathcal{H}_{Q_1})$ and $\mathfrak{S}_{g_{\mathrm{sup}}(E_2)}(\mathcal{H}_{Q_2})$, respectively, it outputs a state whose reduced states are contained in $\mathfrak{S}_{g(E_1,E_2)}(\mathcal{H}_{Q_1})$ and $\mathfrak{S}_{g(E_1,E_2)}(\mathcal{H}_{Q_2})$, respectively, where a function $g:[0,\infty)\times[0,\infty)\to[0,\infty)$ is monotonically increasing and locally bounded in both arguments and satisfies $g(E,E)\leq g_{\mathrm{sup}}(E)$ for any $E\in[0,\infty)$.

The above requirements may be testable for actual noisy gadgets that we can implement in a lab. However, we need to check beforehand whether an s-preparation in Def. 13 can in principle satisfy the E_{prep}-energy constraint and whether an s-parameterized gate in Def. 14 can in principle satisfy the g_{sup}-energy constraint. For an s-preparation gadget, there exists a (pure) state in the form of Eq. (52) with a finite energy, which is shown as follows. Let $s\leq\frac{c}{2}$ and let $F^s(x,y)$ be a finitely supported function that satisfies

$$\forall(x,y):|x|>s\vee|y|>s,\qquad F^s(x,y)=0, \tag{168}$$

$$\iint_{\mathbb{R}} dxdy\,F^s(x,y)=N_s, \tag{169}$$

where N_s is a constant. Then, a state $|\rho^s[\psi]\rangle$ that approximates the GKP state $|\overline{\psi}\rangle=\alpha\,|\overline{0}\rangle+\beta\,|\overline{1}\rangle$ in the sense of Eq. (52) is given by

$$|\rho^s[\psi]\rangle := \frac{1}{\sqrt{N_s}}\iint_{-\infty}^{\infty} dz_1dz_2\,F^s(z_1,z_2)(\alpha\,|0;z_1,z_2\rangle_{LS}+\beta\,|1;z_1,z_2\rangle_{LS}) \tag{170}$$

$$\begin{aligned}&=\frac{1}{\sqrt{cN_s}}\int_{-\infty}^{\infty}dx\iint_{-s}^{s}dz_1dz_2\,e^{iz_1z_2/2}\\&\quad\times\sum_{m\in\mathbb{Z}}e^{2ciz_2m}F^s(z_1,z_2)(\alpha\delta(z_1+2mc-x)+\beta e^{ciz_2}\delta(z_1+(2m+1)c-x)\,|x\rangle_q\,,\end{aligned} \tag{171}$$

where the second equality follows from Eqs. (14) and (23). Here, we can decompose any $x\in\mathbb{R}$ into the nearest multiple of c plus a remainder [8]:

$$x=\lfloor x\rceil_c+\{x\}_c. \tag{172}$$

and we now define the unique integer Then, noticing $\lfloor x\rceil_c/c=\lfloor x/c\rceil$, we have

$$|\rho^s[\psi]\rangle=\frac{1}{\sqrt{cN_s}}\int_{-\infty}^{\infty}dx\int_{-s}^{s}dz_2\,\gamma(\lfloor x/c\rceil)e^{i(x+c\lfloor x/c\rceil)z_2/2}F^s(x-c\lfloor x/c\rceil,z_2)\,|x\rangle_q \tag{173}$$

where $\gamma(n)$ with $n\in\mathbb{Z}$ is defined as

$$\gamma(n):=\begin{cases}\alpha & (n\text{ is even}),\\ \beta & (n\text{ is odd}).\end{cases} \tag{174}$$

Since F^s is supported only on $[-s,s)\times[-s,s)\subseteq\left[-\frac{c}{2},\frac{c}{2}\right)\times\left[-\frac{c}{2},\frac{c}{2}\right)$, we have, for any function $g(x,\lfloor x/c\rceil)$ of x and for any $y\in[-s,s)$,

$$\int dx\,F^s(x-c\lfloor x/c\rceil,y)g(x,\lfloor x/c\rceil)=\int dx\sum_{n\in\mathbb{Z}}F^s(x-cn,y)g(x,n), \tag{175}$$

and

$$\int_{-s}^{s}dz_2\,F^s(y,z_2)e^{iz_2(x+a)/2}=\int_{-\infty}^{\infty}dz_2\,F^s(y,z_2)e^{iz_2(x+a)/2}=\sqrt{2\pi}\,\mathcal{F}_{z_2}[F^s]\Big(y,\frac{x+a}{2}\Big), \tag{176}$$

where $\mathcal{F}_{z_2}[F]$ for a function $F(z_1,z_2)$ denotes the Fourier transform for the second variable. Applying these to Eq. (171), we have

$$|\rho^s[\psi]\rangle=\sqrt{\frac{2c}{N_s}}\int dx\sum_{n\in\mathbb{Z}}\gamma(n)\,\mathcal{F}_{z_2}[F^s]\Big(x-cn,\frac{x+cn}{2}\Big)\,|x\rangle_q \tag{177}$$

$$=\sqrt{\frac{2c}{N_s}}\int dx\,\gamma(\lfloor x/c\rceil)\,\mathcal{F}_{z_2}[F^s]\Big(\{x\}_c,\frac{x+\lfloor x\rceil_c}{2}\Big)\,|x\rangle_q \tag{178}$$

$$= \sqrt{\frac{2c}{N_s}} \int dx \sum_{m\in\mathbb{Z}} \left(\alpha\, \mathcal{F}_{z_2}[F^s]\Big(x - 2mc, \frac{x+2mc}{2}\Big) + \beta\, \mathcal{F}_{z_2}[F^s]\Big(x-(2m+1)c, \frac{x+(2m+1)c}{2}\Big)\right) |x\rangle_q . \quad (179)$$

The momentum wave function has a similar form to the above position wave function due to the symmetry in the subsystem decomposition in Eq. (170). This state has a finite energy as long as $\mathcal{F}_{z_2}[F^s](t,x)$ decays faster than $x^{-3/2}$ for x so that $\langle\rho^s[\psi]|\hat{x}^2|\rho^s[\psi]\rangle$ stays finite. An example of such a function F^s is a product $f^s(z_1)f^s(z_2)$ of bump functions $f^s(z)$ given by

$$f^s(z) \coloneqq \begin{cases} \exp\left(-\frac{1}{1-(x/s)^2}\right) & |x| < s, \\ 0 & \text{otherwise.} \end{cases} \quad (180)$$

Then, the large-scale behavior of $\mathcal{F}_{z_2}[F^s](t,x) = f^s(t)\tilde{f}^s(x)$ is determined by the function $\tilde{f}^s$, where $\tilde{f}^s$ denotes the Fourier transform of f^s. Since the asymptotic behavior of $\tilde{f}^s$ is known to be $\sim |k|^{-\frac{3}{4}}e^{-\sqrt{s|k|}}$ [36] and thus decays faster than any polynomial, this s-parameterized approximate GKP state $|\rho^s[\psi]\rangle$ has a finite energy. (Recall that the energy of a quantum state is given by the sum of the second moments of the position and momentum probability distributions.) Therefore, an s-preparation gadget that prepares the quantum state illustrated above—or any mixtures thereof—can satisfy the E_{prep}-energy constraint for some sufficiently large yet constant E_{prep}. Note that, with the same reasoning, a state prepared as above but with f^s replaced by

$$f^s_{c_1,c_2}(x) \coloneqq \begin{cases} \exp\left(-\frac{c_2}{(1-(x/s)^2)^{c_1}}\right) & |x| < s, \\ 0 & \text{otherwise,} \end{cases} \quad (181)$$

for any $c_1, c_2 > 0$, also has a finite energy [36].

Another example is a smoothly filtered conventional Gaussian approximation of the GKP state, i.e., a (non-normalized) state $\hat{A}^\delta_s |\overline{0}^{\text{app}}_{\sigma^2}\rangle$ for $0 < \delta < s$, which is similar to $\hat{\Pi}_s |\overline{0}^{\text{app}}_{\sigma^2}\rangle$ but has a smooth cutoff on its support instead of a hard cutoff and defined as follows:

$$\hat{A}^\delta_s |\overline{0}^{\text{app}}_{\sigma^2}\rangle \coloneqq \iint_{-\frac{c}{2}}^{\frac{c}{2}} dz_1 dz_2\, \mathrm{PT}[s;\delta](z_1)\, \mathrm{PT}[\mathrm{s},\delta](z_2)\ |0;z_1,z_2\rangle\langle 0;z_1,z_2|_{LS} |\overline{0}^{\text{app}}_{\sigma^2}\rangle , \quad (182)$$

where $\mathrm{PT}[s;\delta]$ denotes the Planck-Taper window function [37] defined as

$$\mathrm{PT}[s;\delta](x) = \begin{cases} 0 & |x| \geq s, \\ \left(1 + \exp\left[\frac{\delta}{s-x} - \frac{\delta}{x-s+\delta}\right]\right)^{-1} & s-\delta < x < s, \\ 1 & |x| \leq s-\delta. \end{cases} \quad (183)$$

The reason we adopt the Planck-Taper window here is that its Fourier transform has a superpolynomial decay [37]. As mentioned above, the large-scale behavior of a wave function is determined by the partial Fourier transform of Eq. (182). Since the Fourier transform of a product of functions is a convolution of Fourier-transformed functions, and the variance of a convolution of two functions is a summation of their variances, the position wave function square of a state $\|\hat{A}^\delta_s |\overline{0}^{\text{app}}_{\sigma^2}\rangle\|^{-1/2}\hat{A}^\delta_s |\overline{0}^{\text{app}}_{\sigma^2}\rangle$ has a finite variance. The same applies to the momentum wave function, and thus the energy of $\|\hat{A}^\delta_s |\overline{0}^{\text{app}}_{\sigma^2}\rangle\|^{-1/2}\hat{A}^\delta_s |\overline{0}^{\text{app}}_{\sigma^2}\rangle$ is finite for $\delta > 0$. We can see that $\|\hat{A}^\delta_s |\overline{0}^{\text{app}}_{\sigma^2}\rangle\|^{-1/2}\hat{A}^\delta_s |\overline{0}^{\text{app}}_{\sigma^2}\rangle \to \|\hat{\Pi}_s |\overline{0}^{\text{app}}_{\sigma^2}\rangle\|^{-1/2}\hat{\Pi}_s |\overline{0}^{\text{app}}_{\sigma^2}\rangle$ as $\delta \to 0$, while its energy diverges in this limit. Thus, there should be a tradeoff between the energy of an s-parameterized GKP state to be compared and the parameter ϵ of (s,ϵ)-independent Markovian noise for a given noisy prepared state. The estimate of the noise strength given in Fig. 5 in Methods is thus optimistic since it computes the trace distance to an infinite-energy state $\|\hat{\Pi}_s |\overline{0}^{\text{app}}_{\sigma^2}\rangle\|^{-1/2}\hat{\Pi}_s |\overline{0}^{\text{app}}_{\sigma^2}\rangle$. The residual term to be evaluated is given by the trace distance between $\|\hat{A}^\delta_s |\overline{0}^{\text{app}}_{\sigma^2}\rangle\|^{-1/2}\hat{A}^\delta_s |\overline{0}^{\text{app}}_{\sigma^2}\rangle$ and $\|\hat{\Pi}_s |\overline{0}^{\text{app}}_{\sigma^2}\rangle\|^{-1/2}\hat{\Pi}_s |\overline{0}^{\text{app}}_{\sigma^2}\rangle$, which may be roughly proportional to δ when $\delta \ll 1$. How small δ can be depends on how large one allows for E_{prep}. We leave a tight evaluation of the noise strength, i.e., the evaluation of the trace distance to $\|\hat{A}^\delta_s |\overline{0}^{\text{app}}_{\sigma^2}\rangle\|^{-1/2}\hat{A}^\delta_s |\overline{0}^{\text{app}}_{\sigma^2}\rangle$, for future work.

Below, we will also prove that the ideal gates given in Supplementary Note 2 A satisfy the g_{sup}-energy constraint with a positive, monotonically increasing, locally bounded function g_{sup}. First, since a phase rotation does not change the energy of a mode, the gate $\hat{F}$ satisfies the g_{sup}-energy constraint as long as $E \leq g_{\text{sup}}(E)$. Next, the displacement operator $\hat{V}(a,b)$ acts on the number operator $\hat{n}$ in the Heisenberg picture as

$$\hat{V}(a,b)^\dagger \hat{n} \hat{V}(a,b) = \hat{V}(a,b)^\dagger \frac{\hat{q}^2+\hat{p}^2-1}{2} \hat{V}(a,b) \quad (184)$$

$$= \frac{(\hat{q}+a)^2 + (\hat{p}+b)^2 - 1}{2} \tag{185}$$

$$= \hat{n} + \frac{a^2+b^2}{2} + a\hat{q} + b\hat{p}. \tag{186}$$

Thus, for any state $\hat{\rho} \in \mathfrak{S}_E(\mathcal{H}_Q)$, we have

$$\langle \hat{V}(a,b)^\dagger \hat{n} \hat{V}(a,b) \rangle_{\hat{\rho}} = E + \frac{a^2+b^2}{2} + a \langle \hat{q} \rangle_{\hat{\rho}} + b \langle \hat{p} \rangle_{\hat{\rho}} \tag{187}$$

$$\leq E + \frac{a^2+b^2}{2} + |a| \sqrt{\langle \hat{q}^2 \rangle_{\hat{\rho}}} + |b| \sqrt{\langle \hat{p}^2 \rangle_{\hat{\rho}}} \tag{188}$$

$$\leq E + \frac{a^2+b^2}{2} + (|a|+|b|)\sqrt{2E+1} \tag{189}$$

Thus, the displacements $\hat{V}(c,0)$ and $\hat{V}(0,c)$ satisfy the g_{sup}-energy constraint as long as

$$g_{\mathrm{sup}}(E) \geq E + c\sqrt{2E+1} + \frac{c^2}{2}. \tag{190}$$

Finally, the SUM gate transforms the number operator $\hat{n}_1$ on the controlled system as

$$\hat{\mathrm{SUM}}^\dagger \hat{n}_1 \hat{\mathrm{SUM}} = \hat{\mathrm{SUM}}^\dagger \frac{\hat{q}^2 + \hat{p}^2 - 1}{2} \hat{\mathrm{SUM}} \tag{191}$$

$$= \frac{\hat{q}_1^2 + (\hat{p}_1 + \hat{p}_2)^2 - 1}{2} \tag{192}$$

$$= \hat{n}_1 + \hat{p}_1 \hat{p}_2 + \frac{\hat{p}_2^2}{2}. \tag{193}$$

We thus have, for any $\hat{\rho} \in \mathfrak{S}(\mathcal{H}_{Q_1 Q_2})$ with $\mathrm{Tr}_{Q_2}[\hat{\rho}] \in \mathfrak{S}_{E_1}(\mathcal{H}_{Q_1})$ and $\mathrm{Tr}_{Q_1}[\hat{\rho}] \in \mathfrak{S}_{E_2}(\mathcal{H}_{Q_2})$, that

$$\langle \hat{\mathrm{SUM}}^\dagger \hat{n}_1 \hat{\mathrm{SUM}} \rangle_{\hat{\rho}} \leq E_1 + \sqrt{\langle \hat{p}_1^2 \rangle_{\hat{\rho}} \langle \hat{p}_2^2 \rangle_{\hat{\rho}}} + \frac{\langle \hat{p}_2^2 \rangle_{\hat{\rho}}}{2} \tag{194}$$

$$\leq E_1 + E_2 + \sqrt{(2E_1+1)(2E_2+1)} + \frac{1}{2}, \tag{195}$$

where the first inequality comes from the positivity of the covariance matrix. The same inequality holds for $\hat{n}_2$, and thus the SUM gate satisfies g_{sup}-energy constraint as long as

$$g_{\mathrm{sup}}(E) \geq 4E + \frac{3}{2}. \tag{196}$$

Thus, we conclude that each ideal gate operation given in Supplementary Note 1 B actually satisfies the g_{sup}-energy constraint defined above for an appropriate function g_{sup}. (In the case of the gate set given above, the function $g_{\mathrm{sup}}(E) = 4E + c\sqrt{2E+1} + \frac{c^2}{2}$, for example, satisfies all conditions.) Notice that $g_{\mathrm{sup}}(E) \geq E$ should always hold for any choice of a gate set since the wait does not change the energy.

For an s-gate to satisfy the energy constraint, the noise channel $\mathcal{N}^s$ also needs to obey the energy constraint. As illustrated in Eq. (189), an up-to-s random displacement channel obeys the g_{sup}-energy constraint with an appropriate function g_{sup}. Thus, there exists a family of channels of the forms in Eq. (99) that obey the g_{sup}-energy constraints. From these, we find that, for any $s > 0$, there exists an s-gate gadget that satisfies the g_{sup}-energy constraint condition with an appropriate function g_{sup}.

Now, we show that the energy of a quantum state during computation is kept finite regardless of the depth D of the circuit. Since we use Knill-type EC gadgets, quantum information is kept teleported to a newly prepared state at every EC gadget. More precisely, we have the following for the output state of an EC gadget.

Proposition 23 (Energy reset of an EC gadget)**.** Let M denote the classical register to keep the GKP Pauli measurement outcomes $\{0,1\}^2$ in a GKP EC gadget given in Eq. (129), and let $\mathcal{P}$ denote the set of probability distributions over $\{0,1\}^2$. For an EC gadget defined in Eq. (129) with the input and output systems labeled by Q_{in} and Q_{out}, respectively, let $\Phi^{\mathrm{EC}} : \mathfrak{S}(\mathcal{H}_{Q_{\mathrm{in}}}) \to \mathcal{P}_M \otimes \mathfrak{S}(\mathcal{H}_{Q_{\mathrm{out}}})$ be a CPTP map of the EC gadget with all the preparation gadgets inside satisfying the E_{prep}-energy constraints and all the gate gadgets inside satisfying the g_{sup}-energy constraints. Then, for any state $\hat{\rho} \in \mathfrak{S}(\mathcal{H}_{Q_{\mathrm{in}}R})$ between the input of the EC gadget and a reference system, $\Phi^{\mathrm{EC}} \otimes \mathrm{Id}_R(\hat{\rho})$ is

a classical-quantum-quantum state between the systems M, Q_{out}, and R with its reduced density operator on the system Q_{out} contained in $\mathfrak{S}_{g_{\mathrm{sup}}^4(E_{\mathrm{prep}})}(\mathcal{H}_{Q_{\mathrm{out}}})$, where g_{sup}^m for $m \in \mathbb{N}$ is defined as

$$g_{\mathrm{sup}}^m \coloneqq \underbrace{g_{\mathrm{sup}} \circ g_{\mathrm{sup}} \circ \cdots \circ g_{\mathrm{sup}}}_{m}. \tag{197}$$

Proof. The output quantum system of Φ^{EC} is generated by the E_{prep}-energy-constrained preparation and goes under four g_{sup}-energy-constrained gate gadgets as shown in Eq. (129). (The number of gates that the prepared state experiences is three, but the first SUM gate takes the maximum energy of the two input states when computing the energy increase.) Thus, the reduced density operator on the system Q_{out} is contained in $\mathfrak{S}_{g_{\mathrm{sup}}^4(E_{\mathrm{prep}})}(\mathcal{H}_{Q_{\mathrm{out}}})$. Since the reduced density operator on Q_{out} is not affected by any other operations in Φ^{EC}, nor by the input state $\hat{\rho}$, the statement holds. Q.E.D.

Therefore, in an FT-GKP circuit with Knill EC gadgets, a quantum state prepared at some point in the circuit experiences only a finite number of gate gadgets before it goes into a measurement gadget. Thus, we arrive at the following conclusion, which is one of the main reasons why we adopt Knill-type error correction in a fault-tolerant GKP EC gadget (although analogous arguments may hold for Steane-type, as well).

Proposition 24. Let ℓ be the maximum number of gate gadgets (including the wait) that a quantum state prepared during computation undergoes before it is measured. Assume that all the preparation gadgets and all the gate gadgets in an FT-GKP circuit satisfy the E_{prep}-energy constraint and the g_{sup}-energy constraint, respectively. Then, a quantum state in the FT-GKP circuit reduced to an arbitrary single mode is always contained in $\mathfrak{S}_{g_{\mathrm{sup}}^{\ell}(E_{\mathrm{prep}})}$ (except inside a measurement gadget, which we need not specify). Furthermore, a reduced density operator of the input of a GKP EC gadget in an FT-GKP circuit is contained in $\mathfrak{S}_{g_{\mathrm{sup}}^{\ell-1}(E_{\mathrm{prep}})}$.

Proof. The first half of the statement is trivial from the definition of ℓ and the energy constraint condition in Defs. 21 and 22. The latter statement follows from the fact that an input state of a GKP EC gadget experiences exactly one g_{sup}-energy-constrained gate as shown in Eq. (129), Q.E.D.

D. Threshold theorem for CV fault-tolerant quantum computation

What we will show in this section is that the noise model defined for a CV circuit in Supplementary Note 1 D will be translated into a local Markovian noise model on the logical qubit circuit, which will be defined below. To describe this noise model, let us consider a quantum circuit consisting of qubits for now. Let $\{C_i\}_{i\in\mathcal{I}}$ be a chronologically ordered set of locations (preparations, gates, measurements, and waits) within a W-qubit D-depth quantum circuit C. (Among the locations at the same time step in C, their order is arbitrary.) Then, $|\mathcal{I}| \leq WD$ holds by definition. Let $\mathcal{O}_i$ be a CPTP map that we want to implement at location C_i. Let $\tilde{\mathcal{O}}_i$ be its noisy version that we can implement in an actual quantum circuit, and assume that it is independent of noise at the other locations. Then, a noisy state $\hat{\rho}^{\mathrm{noisy}}$ implemented by the actual quantum circuit can be written as

$$\hat{\rho}^{\mathrm{noisy}} = \tilde{\mathcal{O}}_{|\mathcal{I}|} \circ \cdots \circ \tilde{\mathcal{O}}_1, \tag{198}$$

$$= (\mathcal{O}_{|\mathcal{I}|} + \mathcal{F}_{|\mathcal{I}|}) \circ \cdots \circ (\mathcal{O}_1 + \mathcal{F}_1), \tag{199}$$

where $\mathcal{F}_i \coloneqq \tilde{\mathcal{O}}_i - \mathcal{O}_i$ is regarded as a fault. There are several important things to note about this formula. First, notice that there is no input state because some of the $\tilde{\mathcal{O}}_i$ are expected to be state preparations. Second, the output $\hat{\rho}^{\mathrm{noisy}}$ may be a quantum state or, if all qubits are measured, a probability distribution. Since the latter can be written as a diagonal density operator, we can use one symbol for both cases (or for a combination thereof). Finally, note that $\mathcal{F}_i$ is not a quantum operation; it is merely a linear map.

When we expand all the parentheses in the above, each term (except for $\mathcal{O}_{|\mathcal{I}|} \circ \cdots \circ \mathcal{O}_1$) is called a *fault path*. In each fault path, the faults $\{\mathcal{F}_i\}$ are applied at certain locations, and the ideal operations $\{\mathcal{O}_i\}$ are applied at all the remaining locations. Let us define the *noise strength* ϵ_i for a particular fault $\mathcal{F}_i$ at location C_i as

$$\epsilon_i \coloneqq \|\mathcal{F}_i\|_\diamond = \|\tilde{\mathcal{O}}_i - \mathcal{O}_i\|_\diamond. \tag{200}$$

The above describes the case of *independent Markovian* noise, where a fault at a given location does not depend on faults in the other locations. *Local Markovian* noise is more general than this. For local Markovian noise, a fault at a given location may depend on faults in other locations as long as the correlations between faults are restricted so that the following definition holds [38].

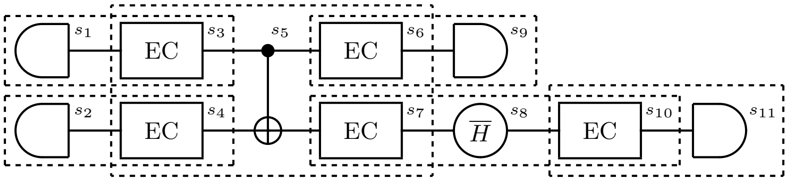

Supplementary Figure 1. Fault-tolerant circuit with extended rectangles (ExRecs) surrounded by broken lines [13].

Definition 25 (Local Markovian noise model)**.** A quantum circuit on qubits and environmental system is said to experience a local Markovian noise model if the noisy evolution can be expanded as a sum of fault paths, where faults are described as the difference between a noisy map (acting as a correlated map on qubits and environmental system) and an ideal map (acting as a tensor-product map on qubits and environmental system), and the norm of a fault path with faults in a set R of specific locations is upper-bounded by $\epsilon_{\text{qubit}}^{|R|}$ for a constant upper bound ϵ_{qubit} on the noise strength at any location.

This type of noise is only approximately Markovian (despite the name). In fact, the definition shows that each fault $\mathcal{F}_i$ can depend on noisy operations in other locations as long as the norm of any fault path falls off exponentially with the number of faults in the path. In Ref. [38], it is shown that local Markovian noise at the kth level of concatenation is translated to local Markovian noise at the $(k+1)^{\text{th}}$ level of concatenation with a different noise strength in a qubit concatenated code.

If a noise model is local Markovian in this sense, then we can bound the total variation distance between a noisy outcome and an ideal one. Let $\hat{\rho}^{\text{ideal}}$ be a density operator representing a classical probability distribution of the measurement outcomes at the end of the circuit given by

$$\hat{\rho}^{\text{ideal}} := \mathcal{O}_{|\mathcal{I}|} \circ \cdots \circ \mathcal{O}_1, \tag{201}$$

and let $\hat{\rho}^{\text{fault}}$ be

$$\hat{\rho}^{\text{fault}} := \hat{\rho}^{\text{noisy}} - \hat{\rho}^{\text{ideal}}. \tag{202}$$

(Note that $\hat{\rho}^{\text{fault}}$ is not a density operator.) Then, the trace distance (or the total variation distance for classical probabilities) between the noisy and the ideal outcome is given by the trace norm of $\hat{\rho}^{\text{fault}}$:

$$\frac{1}{2}\|\hat{\rho}^{\text{noisy}} - \hat{\rho}^{\text{ideal}}\|_1 = \frac{1}{2}\|\hat{\rho}^{\text{fault}}\|_1. \tag{203}$$

The trace norm of $\hat{\rho}^{\text{fault}}$ can be bounded from above when $\epsilon_{\text{qubit}} \leq 1/|\mathcal{I}|$ by the inclusion-exclusion formula [38]. The basic idea is that there are $\binom{|\mathcal{I}|}{r}$ ways of choosing exactly r faulty locations among the $|\mathcal{I}|$ possibilities. Since an upper bound on the norm of a fault path in a local Markovian noise model depends only on the number of faulty locations, we have

$$\|\hat{\rho}^{\text{fault}}\|_1 \leq \left\| \sum_{r=1}^{|\mathcal{I}|} [\text{fault path with } r \text{ faulty locations}] \right\|_1 \leq \sum_{r=1}^{|\mathcal{I}|} \binom{|\mathcal{I}|}{r} \epsilon_{\text{qubit}}^r \leq (1+\epsilon_{\text{qubit}})^{|\mathcal{I}|} - 1 \leq (e-1)|\mathcal{I}|\, \epsilon_{\text{qubit}}, \tag{204}$$

where we used in the last inequality that $(1+x)^a - 1 \leq \frac{(1+x_{\max})^a - 1}{x_{\max}} x$ holds for $a \geq 1$ and $x \in [0, x_{\max}]$, and that $(1+1/x)^x$ for $x > 0$ is monotonically increasing and approaches e.

We aim to show the level reduction from a CV quantum circuit with a noise model defined in Supplementary Note 1 D to a qubit quantum circuit with a local Markovian noise model. For this, we define the following.

Definition 26. A GKP extended rectangle (GKP ExRec) in a fault-tolerant circuit C' consists of an FT-GKP gadget C'_i replacing a location C_i of an original qubit circuit C plus all the FT-GKP EC gadgets between C'_i and the gadgets adjacent to it in the circuit C'. The FT-GKP EC steps before C'_i are called leading FT-GKP EC steps, and those after C'_i are called trailing FT-GKP EC steps.

The GKP ExRecs in the circuit are illustrated in Supplementary Figure 1. We want to reduce the question of whether a higher-level (qubit-level) circuit is perfect or erroneous to an equivalent question of whether its constituent GKP ExRecs are correct or not, where the correctness of a GKP ExRec is defined below. Our conditions for correctness are

formally the same as those in Ref. [13], while the meanings of gadgets and the ideal decoder are different. Importantly, we have introduced the appropriate equivalence class of noise with the gadgets, filter, and the ideal decoder for the GKP code so that we can repurpose the techniques developed for proving a threshold theorem with a qubit concatenated code in Ref. [13] for the rest of our analysis.

Definition 27 (Correctness for the ideal GKP decoder)**.** A GKP gate or wait ExRec is correct if

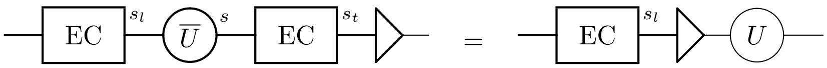

(205)

A GKP-preparation ExRec is correct if

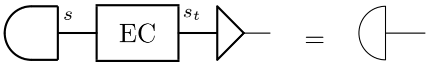

(206)

A GKP-measurement ExRec is correct if

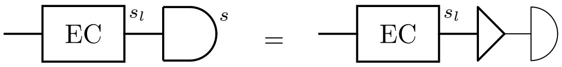

(207)

We also define the goodness/badness of a GKP ExRec as has been done in Ref. [13].

Definition 28 (Good or bad GKP ExRec)**.** A GKP preparation ExRec is good if it consists of an s-preparation gadget followed by an s_t-EC gadget satisfying $s + s_t < \frac{c}{2}$. A GKP single-qubit gate ExRec is good if it consists of a leading s_l-EC gadget, an s-gate, and a trailing s_t-EC gadget satisfying $s_l + s + s_t < \frac{c}{2}$. A GKP two-qubit gate ExRec is good if it consists of leading s_l-EC gadgets, an s-gate, and trailing s_t-EC gadgets satisfying $2s_l + s + s_t < \frac{c}{2}$. A GKP measurement ExRec is good if it consists of an s_l-EC gadget followed by an s-measurement gadget satisfying $s_l + s < \frac{c}{2}$. A GKP ExRec is called bad if it is not good.

Using the same argument as the qubit case in Ref. [13], we see that the goodness of the GKP ExRec is a sufficient condition for its correctness, as shown in the following lemma.

Lemma 29 (Good implies correct for the ideal GKP decoder)**.** If a GKP ExRec is good, then it is correct for the ideal GKP decoder.

Proof. Let us use a good GKP single-qubit gate ExRec as an example, and the other cases can be proved with the same reasoning. From Proposition 19 and 20, the following chain of equalities holds.

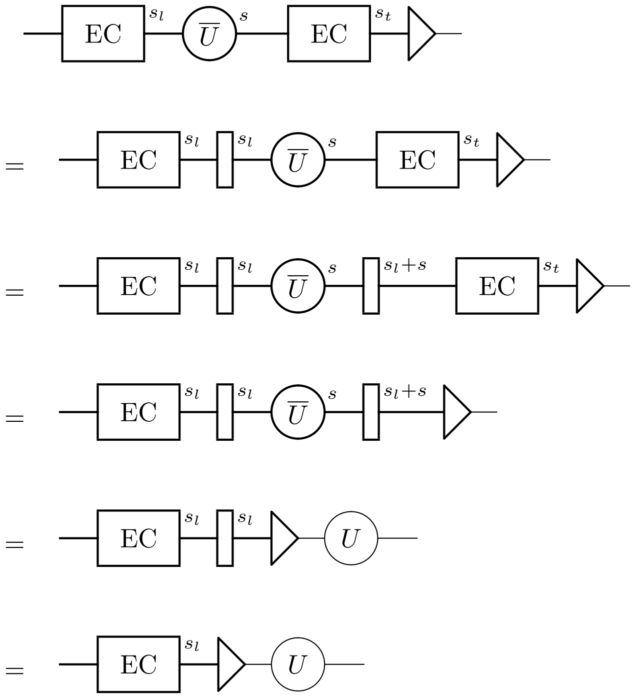

In the above, we used the fact that $s_l + s + s_t < \frac{c}{2}$, which is from the goodness of this ExRec. Thus, we reach the correctness of the ExRec. Q.E.D.

It is thus obvious that if an FT-GKP circuit contains only good GKP ExRecs, the output distribution of the FT protocol C' with all the measurement outcomes of EC gadgets ignored is the same as the output distribution of the qubit circuit C. Even though it is unlikely that all the GKP ExRecs are good, we can use a qubit concatenated code to suppress errors at a higher level of concatenation so that the frequency of bad ExRecs at the concatenation level k is arbitrarily small [13]. To show this, however, we need to specify how a bad ExRec should be interpreted in a higher-level (qubit-level) circuit. Unfortunately, the corresponding logical qubit picture for a bad GKP ExRec cannot be determined locally since it may depend on leftover errors in the previous ExRecs. We thus need to see larger contexts to determine the erroneous logical qubit operation. For this, we introduce the GKP $*$-decoder $\mathcal{D}^*_{\mathrm{GKP}}$ by generalizing the $*$-decoder in Ref. [13]. The GKP $*$-decoder keeps the syndrome subsystem $\mathcal{H}_S$ instead of tracing it out as is done in the ideal decoder Eq. (131), i.e.,

$$\mathcal{D}^*_{\mathrm{GKP}}(\hat{\rho}) = \sum_{\mu,\nu\in\{0,1\}} \iint_{|z_1|,|z_2|<\frac{c}{2}} dz_1 dz_2 \iint_{|z_1'|,|z_2'|<\frac{c}{2}} dz_1' dz_2' \, |\mu; z_1, z_2\rangle\langle\mu; z_1, z_2|_{LS} \, \hat{\rho} \, |\nu; z_1', z_2'\rangle\langle\nu; z_1', z_2'|_{LS} \, . \tag{208}$$

One can check from Eq. (28) that $\mathcal{D}^*_{\mathrm{GKP}}$ is nothing but a basis transform, representing a state in the SSS Hilbert space. Diagrammatically, the GKP $*$-decoder is denoted as follows.

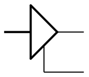

GKP $*$-decoder

In this diagram, the line at the top denotes the logical qubit L and the line at the bottom denotes the syndrome subsystem S. In this sense, the stabilizer subsystem decomposition [1] is nothing but the $*$-decoder in our analysis. For a bad GKP-gate ExRec, we thus have the following.

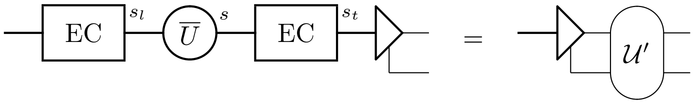

(209)

The noisy interaction (channel) $\mathcal{U}'$ depends on the gate $\overline{U}$ and noise during the ExRec. This time, the GKP $*$-decoder absorbs the entire GKP ExRec (including the leading EC gadgets, unlike good GKP ExRecs where the decoder leaves the leading EC gadgets as shown in Lemma 29) to avoid correlated errors between consecutive gates in C. Similar relations hold also for bad GKP-measurement ExRecs and preparation ExRecs. For a bad GKP-measurement ExRec, a qubit Pauli-Z/X measurement is performed on the logical qubit L while the syndrome subsystem S is traced out just after a noise operator entangles two subsystems L and S. For a bad GKP-preparation ExRec, a noise operator entangles the logical qubit L and the syndrome subsystem S immediately after the preparation of a logical qubit state in $\mathcal{H}_L$ and an arbitrary state in $\mathcal{H}_S$, or equivalently, an entangled state in $\mathcal{H}_L \otimes \mathcal{H}_S$ is prepared. Note that the form of $\mathcal{U}'$ implicitly assumes the independence and the Markovianity of noise. Otherwise, $\mathcal{U}'$ may act on several locations at the same time (if not independent) and may depend on an environmental state (if not Markovian). The same applies to the following arguments.

Now, we need to redefine the correctness of a GKP ExRec for the GKP $*$-decoder as follows.

Definition 30 (Correctness for the GKP $*$-decoder)**.** A GKP gate ExRec is correct if it satisfies the following.

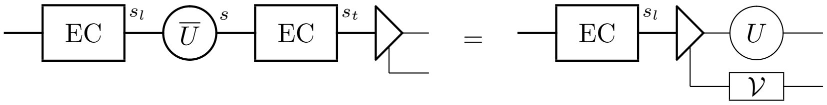

(210)

In the above, $\mathcal{V}$ is a CPTP map acting only on the syndrome subsystem S, which depends on the gate $\overline{U}$ and the noise during the ExRec. Based on this principle, analogous definitions extending Definitions 27 exist for a correct GKP-measurement ExRec and a correct GKP-preparation ExRec, as well.

The important thing in the above definition is that the correct logical gate $\hat{U}$ is applied and no correlations are introduced between the logical qubit and the syndrome subsystem. For a good GKP ExRec, we have the following.

Lemma 31 (Good implies correct for the $*$-decoder)**.** If a GKP ExRec is good, then it is also correct for the $*$-decoder.

Proof. Notice that the ideal GKP decoder corresponds to the GKP $*$-decoder with the syndrome subsystem S ignored. Since the goodness of a GKP ExRec implies its correctness for the ideal GKP decoder, the logical subsystem L must be decoupled from the syndrome subsystem S. Then, it can be seen that Eq. (210) is the most general form of such decoupled dynamics. Q.E.D.

So far, we have seen how good and bad ExRecs can be interpreted in the qubit picture. However, since we forced the GKP $*$-decoder to erase all the gadgets within a bad GKP ExRec, the leading consecutive ExRec lacks the trailing GKP EC gadget. Thus, we need the following definition.

Definition 32 (Truncated GKP ExRec)**.** A GKP ExRec missing one or more trailing ECs is called a truncated GKP ExRec (except for GKP measurement ExRecs). A truncated GKP gate ExRec is correct for the GKP $*$-decoder (and thus for the ideal GKP decoder) if it satisfies the following.

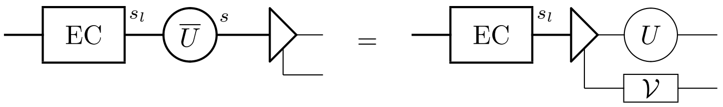

(211)

We can similarly define the correctness for a truncated GKP preparation ExRec.

In the same way as a full GKP ExRec, we define the goodness and badness of a truncated GKP ExRecs.

Definition 33. A truncated GKP preparation ExRec is good if it consists of an s-preparation gadget with $s < \frac{c}{2}$. A truncated GKP gate ExRec is good if it consists of a leading s_l-EC gadget and an s-gate with $s_l + s < \frac{c}{2}$. A truncated GKP ExRec is bad if it is not good.

One can notice that the good GKP ExRec is also good when truncated, which will be used later. One can derive the following with the same procedures as the previous discussions (and thus we omit the proof).

Lemma 34. A good truncated GKP ExRec is correct for the GKP $*$-decoder (and thus for the ideal GKP decoder).

We can now reduce our noisy CV quantum circuit consisting of multiple GKP ExRecs to a qubit-level quantum circuit.

Definition 35 (Noisy quantum circuit defined through the GKP code)**.** Given an FT-GKP CV circuit C' for implementing an ideal qubit circuit C, a circuit $\tilde{C}$ on qubits and environmental systems is defined by moving the GKP $*$-decoder from the end to the start of the circuit C' as in Eqs. (209), (210), and (211) and regarding the syndrome subsystems as environmental systems.

We finally come to the point of stating our level-reduction theorem. Recall that we have defined our noise model using three parameters, i.e., s, ϵ, and E in Defs. 8–10. The essence of our analysis is to take sufficiently small constant s so that the FT-GKP CV circuit C' should be able to simulate the original qubit circuit C correctly in the ideal case (i.e., in the case of $\epsilon = 0$), and then take into account the effect of actual nonzero ϵ in a non-ideal case by perturbatively evaluating the norm of each fault path for $\tilde{C}$. In particular, we choose the parameters s_{p}, s_{g}, s_{m}, and s_{e} to satisfy the following conditions:

$$2s_{\mathrm{p}} + 2s_{\mathrm{g}} + \max\{s_{\mathrm{g}} + s_{\mathrm{m}}, 2s_{\mathrm{g}}\} = s_{\mathrm{e}}, \tag{212}$$

$$s_{\mathrm{p}} + s_{\mathrm{e}} < \frac{c}{2}, \tag{213}$$

$$3s_{\mathrm{e}} + s_{\mathrm{g}} < \frac{c}{2}, \tag{214}$$

$$s_{\mathrm{e}} + s_{\mathrm{m}} < \frac{c}{2}. \tag{215}$$

These conditions ensure that all the GKP preparation ExRecs, gate ExRecs (including the two-mode gate), and measurement ExRecs composed of s_{p}-preparations, s_{g}-gates, and s_{m}-measurements in the circuit C' are good. Then, the evaluation of errors in the qubit circuit $\tilde{C}$ reduces to the evaluation of the deviation from this "idealized" situation in the CV circuit C' due to the effect of nonzero ϵ.

To evaluate this effect, let $E^{\ell}_{\max}(\epsilon)$ be a function of $\epsilon \in (0, 1)$ defined as

$$E^{\ell}_{\max}(\epsilon) := g_{\sup}\left(\frac{g^{\ell-1}_{\sup}(E_{\mathrm{prep}})}{\epsilon^2}\right), \tag{216}$$

where E_{prep} and g_{sup} are a positive constant and a positive, monotonically increasing, locally bounded function, respectively, which define the energy-constraint conditions in Defs. 21 and 22, and ℓ is the maximum number of gate gadgets that a quantum state prepared in the circuit C' undergoes before it is measured as defined in Prop. 24. Notice that $E_{\max}^{\ell}(\epsilon)$ increases when ϵ decreases. Then, we prove the following theorem.

Theorem 36 (Level reduction)**.** Let C' be an FT-GKP circuit on CV systems for implementing a circuit C on qubits. Let E_{prep} and g_{sup} be a positive constant and a positive, monotonically increasing, locally bounded function, respectively. Suppose that all the preparation gadgets and gate gadgets in C' satisfy the E_{prep}-energy constraint and the g_{sup}-energy constraint, respectively (Defs. 21 and 22). Also, for ϵ satisfying $0 < \epsilon < 1$ and s_{p}, s_{g}, and s_{m} satisfying Eqs. (212)–(215), suppose that the CV circuit C' experiences $(s_{\mathrm{p}}, \epsilon)$-independent Markovian noise for state preparations, $(E_{\max}^{\ell}(\epsilon), s_{\mathrm{g}}, \epsilon)$-independent Markovian noise for gates (including waits), and $(E_{\max}^{\ell}(\epsilon), s_{\mathrm{m}}, \epsilon)$-independent Markovian noise for measurements (Defs. 8–10), where an s_{p}-preparation and an s_{g}-gate inside the definitions of these noises also satisfy the E_{prep}-energy constraint and the g_{sup}-energy constraint, respectively. For a nonempty subset $R \subseteq \mathcal{I}$ of the indices of the locations in C, let $\hat{\rho}_R$ be the sum of fault paths of the quantum computation with faults applied to at least one location in the ith truncated GKP ExRec in C' (i.e., corresponding to the location C_i in C) for each $i \in R$, where faults are described in the sense of Eq. (199). For any such sum of fault paths $\hat{\rho}_R$ in C', define a fault path in a circuit $\tilde{C}$ on qubits and environmental systems as follows: replace the location C_i of C for each $i \in R$ with the corresponding fault map acting both on the qubits and the environmental systems in C', which can, for example, be described as the difference between $\mathcal{U}'$ in Eq. (209) and $\mathcal{U} \otimes \mathcal{V}$ in Eq. (210), and keep the others (i.e., C_i for $i \in \mathcal{I} \setminus R$) unchanged on the qubits while acting appropriate channels on the environmental systems as in Eq. (210). Then, the circuit $\tilde{C}$ experiences a local Markovian noise model (Def. 25). Furthermore, an upper bound $\epsilon_{\mathrm{qubit}}$ on the noise strength at any location in the circuit $\tilde{C}$ is given by

$$\epsilon_{\mathrm{qubit}} = 10\epsilon L_{\max}, \tag{217}$$

where $L_{\max}$ denotes the maximum number of GKP preparation, gate, and measurement gadgets in any truncated GKP ExRec in C'.

Proof. Our proof is based on evaluating the effect of nonzero ϵ, applying the techniques in the analysis of a threshold theorem under independent Markovian noise on qubits [38] to the GKP code. Since $E_{\max}^{\ell}(\epsilon)$ is a function of ϵ, the noise model here has two types of parameters; i.e., one is s_{p}, s_{g}, s_{m}, or s_{e}, and the other is ϵ. As is clear from the correctness conditions of GKP ExRecs, the GKP code can tolerate and correct the effect of nonzero s_{p}, s_{g}, s_{m}, and s_{e}; in particular, with s_{p}, s_{g}, s_{m}, and s_{e} satisfying Eqs. (212)–(215), if ϵ were zero, then the FT-GKP circuit C' for C that consists only of s_{p}-preparations, s_{g}-gates, s_{m}-measurements, and s_{e}-ECs would output the same probability distribution as the ideal qubit circuit C since all the GKP ExRecs become good (see Def. 28). By contrast, it turns out that a nonzero ϵ may directly lead to an error on the logical qubit of the GKP code. In this sense, our analysis of the effect of ϵ on the GKP code is analogous to considering a distance-1 code on qubits in the level reduction under an independent Markovian noise model [38]; however, the difference between Ref. [38] and our analysis of GKP code arises since we also need to use bounds on the energy-constrained diamond norm introduced in Supplementary Note 1 C.

In our case, each fault in a fault path of $\tilde{C}$ can be regarded as the difference between an ideal preparation, gate, or measurement in C and the corresponding erroneous one, such as $\mathcal{U}'$ in Eq. (209), which is given by moving the GKP $*$-decoder through the whole circuit as in Def. 35. Thus, one fault in a fault path of the CV circuit C', which is the difference between the actual noisy gate (resp. preparation, measurement) and a corresponding s_{g}-gate (resp. s_{p}-preparation, s_{m}-measurement), may immediately cause a fault in $\tilde{C}$ (which is akin to a distance-1 code on qubits in the sense that no fault is tolerated). In this case, given a fault path, if the trailing EC gadget of a full GKP ExRec includes faults, then these faults are always taken into account as those in the leading EC gadget of the successive GKP ExRec due to the rule in Def. 35, and thus the given GKP ExRec becomes a truncated GKP ExRec; therefore, in our analysis (similar to the analysis of the distance-1 code on qubits), it suffices to count faults in truncated GKP ExRecs. In other words, a fault in $\tilde{C}$ may occur if at least one of the CV preparations, gates, and measurements in the corresponding truncated GKP ExRec (i.e., the leading EC gadgets or the location C_i' corresponding to C_i in C) is not an E_{prep}-energy-constrained s_{p}-preparation, a g_{sup}-energy-constrained s_{g}-gate, and an s_{m}-measurement, respectively. We will thus bound, for a nonempty subset $R \subseteq \mathcal{I}$, the norm of the sum $\hat{\rho}_R$ of fault paths in C' in which faults apply to at least one location in the ith truncated GKP ExRec in C' for each $i \in R$, which then corresponds to a fault path in $\tilde{C}$ where faults apply to $\{\tilde{C}_i\}_{i \in R}$.

Let $\{C_j'\}_{j \in \mathcal{I}'}$ be a chronologically ordered set of locations in C'. (Recall that each element may be a preparation, gate, or measurement.) For brevity in what follows, we define a composite map realized at locations from C_a' to C_b'

by

$$\mathcal{O}_{b:a} \coloneqq \prod_{j=a}^{b} \mathcal{O}_j = \mathcal{O}_b \circ \mathcal{O}_{b-1} \circ \cdots \circ \mathcal{O}_{a+1} \circ \mathcal{O}_a \tag{218}$$

with the composition product taken in an appropriate order, starting with a (on the right) and ending with b (on the left). We will use two version of this definition, $\mathcal{O}^{\text{noisy}}_{b:a}$ and $\mathcal{O}^{\text{ideal}}_{b:a}$, where an individual CPTP map $\mathcal{O}^{\text{noisy}}_j$ (resp. $\mathcal{O}^{\text{ideal}}_j$) with $j \in \mathcal{I}'$ corresponds to either an E_{prep}-energy-constrained actual preparation (resp. s_{p}-preparation), a g_{sup}-energy-constrained actual gate (resp. s_{g}-gate), or an actual measurement (resp. s_{m}-measurement) at the jth location in C'. Let us further define the fault map $\mathcal{F}_j$ as

$$\mathcal{F}_j \coloneqq \mathcal{O}^{\text{noisy}}_j - \mathcal{O}^{\text{ideal}}_j. \tag{219}$$

From the definition of the (s,ϵ)- and (E,s,ϵ)-independent Markovian noise in Defs. 8–10, we have

$$\frac{1}{2}\|\mathcal{F}_j\|_{\diamond}^{E^{\ell}_{\max}(\epsilon)} \le \epsilon, \tag{220}$$

where we slightly abuse the notation when $\mathcal{O}^{\text{noisy}}_j$ and $\mathcal{O}^{\text{ideal}}_j$ are state-preparation maps by defining $\mathfrak{S}_E(\mathbb{C}) \coloneqq \{1\} =: \mathfrak{S}(\mathbb{C})$. Then, the sum $\hat{\rho}_R$ of fault paths defined above is given by

$$\hat{\rho}_R = \sum_{i\in R}\sum_{T_i\subseteq R'_i:|T_i|\ge 1}\big[\text{Fault } \mathcal{F}_j \text{ replaces } \mathcal{O}^{\text{ideal}}_j \text{ in } \mathcal{O}^{\text{ideal}}_{|\mathcal{I}'|:1} \text{ for all } j\in T_i\big], \tag{221}$$

where $R'_i \subseteq \mathcal{I}'$ for $i \in R$ denotes a set of indices that correspond to the locations of ith truncated GKP ExRec.

Let i_{ini} be the initial (smallest) element of the ordered set R and let j_{ini} and j_{fin} be the initial and the final elements of $R'_{i_{\text{ini}}}$, i.e., $R'_{i_{\text{ini}}} = \{j_{\text{ini}},\dots,j_{\text{fin}}\}$. Then, by using the definition of $\mathcal{F}_j$ in Eq. (219), we have

$$\begin{aligned}\hat{\rho}_R &= \mathcal{O}'_{|\mathcal{I}'|:j_{\text{fin}}+1} \circ \left(\sum_{T\subseteq\{j_{\text{ini}},\dots,j_{\text{fin}}\}:|T|\ge 1}\big[\text{Fault } \mathcal{F}_j \text{ replaces } \mathcal{O}^{\text{ideal}}_j \text{ in } \mathcal{O}^{\text{ideal}}_{j_{\text{fin}}:j_{\text{ini}}} \text{ for all } j\in T\big]\right) \circ \mathcal{O}^{\text{ideal}}_{j_{\text{ini}}-1:1} &(222)\\ &= \mathcal{O}'_{|\mathcal{I}'|:j_{\text{fin}}+1} \circ \left(\mathcal{O}^{\text{noisy}}_{j_{\text{fin}}:j_{\text{ini}}} - \mathcal{O}^{\text{ideal}}_{j_{\text{fin}}:j_{\text{ini}}}\right) \circ \mathcal{O}^{\text{ideal}}_{j_{\text{ini}}-1:1}, &(223)\end{aligned}$$

where

$$\mathcal{O}'_{|\mathcal{I}'|:j_{\text{fin}}+1} \coloneqq \sum_{i\in R\setminus\{i_{\text{ini}}\}}\sum_{T_i\subseteq R'_i:|T_i|\ge 1}\big[\text{Fault } \mathcal{F}_j \text{ replaces } \mathcal{O}^{\text{ideal}}_j \text{ in } \mathcal{O}^{\text{ideal}}_{|\mathcal{I}|':j_{\text{fin}}+1} \text{ for all } j\in T_i\big], \tag{224}$$

and $\mathcal{O}_{j:j+1} \coloneqq \text{Id} =: \mathcal{O}_{j-1:j}$ for notational convenience (since, in both cases, the initial index is after the final index). By adding

$$0 = \mathcal{O}'_{|\mathcal{I}'|:j_{\text{fin}}+1} \circ \left(\sum_{j=j_{\text{ini}}}^{j_{\text{fin}}-1} -\mathcal{O}^{\text{noisy}}_{j_{\text{fin}}:j+1} \circ \mathcal{O}^{\text{ideal}}_{j:j_{\text{ini}}} + \mathcal{O}^{\text{noisy}}_{j_{\text{fin}}:j+1} \circ \mathcal{O}^{\text{ideal}}_{j:j_{\text{ini}}}\right) \circ \mathcal{O}^{\text{ideal}}_{j_{\text{ini}}-1:1} \tag{225}$$

to Eq. (223), we have[1]

$$\hat{\rho}_R = \mathcal{O}'_{|\mathcal{I}'|:j_{\text{fin}}+1} \circ \left(\sum_{j=j_{\text{ini}}}^{j_{\text{fin}}} \mathcal{O}^{\text{noisy}}_{j_{\text{fin}}:j+1} \circ \mathcal{F}_j \circ \mathcal{O}^{\text{ideal}}_{j-1:j_{\text{ini}}}\right) \circ \mathcal{O}^{\text{ideal}}_{j_{\text{ini}}-1:1}. \tag{226}$$

[1] Another way to see this is to reconsider the difference $\mathcal{O}^{\text{noisy}}_{j_{\text{fin}}:j_{\text{ini}}} - \mathcal{O}^{\text{ideal}}_{j_{\text{fin}}:j_{\text{ini}}}$ in Eq. (223), which is the sum in parentheses in Eq. (222). The first term, $\mathcal{O}^{\text{noisy}}_{j_{\text{fin}}:j_{\text{ini}}}$, is the sum over all $2^{|T|}$ fault paths, including the no-fault path. The only thing that subtracting the second term, $\mathcal{O}^{\text{ideal}}_{j_{\text{fin}}:j_{\text{ini}}}$, is doing is guaranteeing at least one fault. And this means there must be a location of the *first* fault, which we call j. Now, group the terms in this sum according to this location. By definition, (1) there are no faults before this location ($\mathcal{O}^{\text{ideal}}_{j-1:j_{\text{ini}}}$), (2) $\mathcal{F}_j$ has replaced the ideal operation at this location, and (3) the rest of the locations may or may not have any faults, and we must sum over these possibilities ($\mathcal{O}^{\text{noisy}}_{j_{\text{fin}}:j+1}$). Composing these and summing over all first-fault locations j gives the result in Eq. (226).

Now, we upper-bound the trace norm of $\hat{\rho}_R$. (Recall that $\{\mathcal{O}_j\}_{j\in\mathcal{I}'}$ includes a state-preparation map.) Using $\|\mathcal{U}(\hat{\rho})\|_1 \leq \|\mathcal{U}\|_\diamond^E \|\hat{\rho}\|_1$ for any linear map $\mathcal{U}$ and any state $\hat{\rho} \in \mathfrak{S}_E(\mathcal{H}_Q)$, we have

$$\|\hat{\rho}_R\|_1 = \left\| \mathcal{O}'_{|\mathcal{I}'|:j_{\mathrm{fin}}+1} \circ \left(\sum_{j=j_{\mathrm{ini}}}^{j_{\mathrm{fin}}} \mathcal{O}^{\mathrm{noisy}}_{j_{\mathrm{fin}}:j+1} \circ \mathcal{F}_j \circ \mathcal{O}^{\mathrm{ideal}}_{j-1:j_{\mathrm{ini}}} \right) \circ \mathcal{O}^{\mathrm{ideal}}_{j_{\mathrm{ini}}-1:1} \right\|_1 \tag{227}$$

$$\leq \left\| \mathcal{O}'_{|\mathcal{I}'|:j_{\mathrm{fin}}+1} \circ \left(\sum_{j=j_{\mathrm{ini}}}^{j_{\mathrm{fin}}} \mathcal{O}^{\mathrm{noisy}}_{j_{\mathrm{fin}}:j+1} \circ \mathcal{F}_j \circ \mathcal{O}^{\mathrm{ideal}}_{j-1:j_{\mathrm{ini}}} \right) \right\|_\diamond^{g_{\mathrm{sup}}^{\ell-1}(E_{\mathrm{prep}})} \|\mathcal{O}^{\mathrm{ideal}}_{j_{\mathrm{ini}}-1:1}\|_1 \tag{228}$$

$$\leq \sum_{j=j_{\mathrm{ini}}}^{j_{\mathrm{fin}}} \left\| \mathcal{O}'_{|\mathcal{I}'|:j_{\mathrm{fin}}+1} \circ \left(\mathcal{O}^{\mathrm{noisy}}_{j_{\mathrm{fin}}:j+1} \circ \mathcal{F}_j \circ \mathcal{O}^{\mathrm{ideal}}_{j-1:j_{\mathrm{ini}}} \right) \right\|_\diamond^{g_{\mathrm{sup}}^{\ell-1}(E_{\mathrm{prep}})} \tag{229}$$

$$\leq \sum_{j=j_{\mathrm{ini}}}^{j_{\mathrm{fin}}} \left\| \mathcal{O}'_{|\mathcal{I}'|:j_{\mathrm{fin}}+1} \circ \left(\mathcal{O}^{\mathrm{noisy}}_{j_{\mathrm{fin}}:j+1} \circ \mathcal{F}_j \circ \mathcal{O}^{\mathrm{ideal}}_{j-1:j_{\mathrm{ini}}} \right) \right\|_\diamond^{g_{\mathrm{sup}}^{\ell-1}(E_{\mathrm{prep}})/\epsilon^2}, \tag{230}$$

where the first inequality follows from the fact that an input state of a GKP EC gadget (i.e., an output state of the leading truncated GKP ExRec) is contained in $g_{\mathrm{sup}}^{\ell-1}(E_{\mathrm{prep}})$ as shown in Prop. 24, the second inequality from the triangle inequality and $\|\mathcal{O}^{\mathrm{ideal}}_{j_{\mathrm{ini}}-1:1}\|_1 = 1$, and the last inequality from the monotonicity of the energy-constrained diamond norm under energy increase in Eq. (58). Notice that

$$\mathcal{O}^{\mathrm{noisy}}_{j_{\mathrm{fin}}:j+1} \circ \mathcal{O}^{\mathrm{ideal}}_{j:j_{\mathrm{ini}}} : \mathfrak{S}(\mathcal{H}_Q) \to \mathfrak{S}_{g_{\mathrm{sup}}^{\ell-1}(E_{\mathrm{prep}})}(\mathcal{H}_Q) \tag{231}$$

holds for any $j \in \{j_{\mathrm{ini}}-1, \ldots, j_{\mathrm{fin}}\}$ from Props. 23 and 24. Furthermore, we have that

$$\mathcal{O}^{\mathrm{ideal}}_{j-1:j_{\mathrm{ini}}} : \mathfrak{S}_{g_{\mathrm{sup}}^{\ell-1}(E_{\mathrm{prep}})/\epsilon^2}(\mathcal{H}_Q) \to \mathfrak{S}_{E^\ell_{\mathrm{max}}(\epsilon)}(\mathcal{H}_Q) \tag{232}$$

holds from Eq. (216) and the fact that the input of a GKP EC gadget undergoes only one gate before being measured and that energy of states newly prepared at the GKP EC gadget is bounded from above by $g_{\mathrm{sup}}^{\ell-1}(E_{\mathrm{prep}}) \leq E^\ell_{\mathrm{max}}(\epsilon)$ for any $j \in \{j_{\mathrm{ini}}, \ldots, j_{\mathrm{fin}}\}$. We thus have

$$\left\| \mathcal{O}^{\mathrm{noisy}}_{j_{\mathrm{fin}}:j+1} \circ \mathcal{F}_j \circ \mathcal{O}^{\mathrm{ideal}}_{j-1:j_{\mathrm{ini}}} \right\|_\diamond^{g_{\mathrm{sup}}^{\ell-1}(E_{\mathrm{prep}})/\epsilon^2} \leq \|\mathcal{O}^{\mathrm{noisy}}_{j_{\mathrm{fin}}:j+1} \circ \mathcal{F}_j\|_\diamond^{E^\ell_{\mathrm{max}}(\epsilon)} \|\mathcal{O}^{\mathrm{ideal}}_{j-1:j_{\mathrm{ini}}}\|_\diamond^{g_{\mathrm{sup}}^{\ell-1}(E_{\mathrm{prep}})/\epsilon^2} \leq \|\mathcal{O}^{\mathrm{noisy}}_{j_{\mathrm{fin}}:j+1}\|_\diamond \|\mathcal{F}_j\|_\diamond^{E^\ell_{\mathrm{max}}(\epsilon)} \leq 2\epsilon, \tag{233}$$

where we used Eqs. (59) and (232) in the first inequality, Eq. (60) in the second inequality, and Eq. (220) in the last inequality. Note also that $\|\mathcal{O}\|_\diamond = 1 = \|\mathcal{O}\|_\diamond^E$ holds for any CPTP map $\mathcal{O}$. Then, from Eqs. (219), (231), and (233), we can apply Corol. 6 to the pair $\mathcal{O}^{\mathrm{noisy}}_{j_{\mathrm{fin}}:j} \circ \mathcal{O}^{\mathrm{ideal}}_{j-1:j_{\mathrm{ini}}}$ and $\mathcal{O}^{\mathrm{noisy}}_{j_{\mathrm{fin}}:j+1} \circ \mathcal{O}^{\mathrm{ideal}}_{j:j_{\mathrm{ini}}}$ of CPTP maps in Eq. (230) for any $j \in \{j_{\mathrm{ini}}, \ldots, j_{\mathrm{fin}}\}$ to have

$$\|\hat{\rho}_R\|_1 \leq 10\epsilon(j_{\mathrm{fin}} - j_{\mathrm{ini}} + 1) \|\mathcal{O}'_{|\mathcal{I}'|:j_{\mathrm{fin}}+1}\|_\diamond^{g_{\mathrm{sup}}^{\ell-1}(E_{\mathrm{prep}})/\epsilon^2} \tag{234}$$

$$\leq 10\epsilon L_{\mathrm{max}} \|\mathcal{O}'_{|\mathcal{I}'|:j_{\mathrm{fin}}+1}\|_\diamond^{g_{\mathrm{sup}}^{\ell-1}(E_{\mathrm{prep}})/\epsilon^2}, \tag{235}$$

where we used $j_{\mathrm{fin}} - j_{\mathrm{ini}} + 1 = |R'_{i_{\mathrm{ini}}}| \leq L_{\mathrm{max}}$ from the definition of L_{max}. Here, we notice from Eqs. (230) and Eq. (235) that the same line of argument to obtain an upper bound applies to $i_{\mathrm{ini}+1} \in R$ and so on. Thus, we inductively prove

$$\|\hat{\rho}_R\|_1 \leq (10\epsilon L_{\mathrm{max}})^{|R|}. \tag{236}$$

Therefore, the circuit $\tilde{C}$ experiences the local Markovian noise model with the noise strength upper-bounded by $10\epsilon L_{\mathrm{max}} =: \epsilon_{\mathrm{qubit}}$. Q.E.D.

One may notice that the analysis above does not fully exploit the advantage of using ExRecs since all the ExRecs just become good if ϵ is zero. It is also possible to consider a more general setting where parameters s_{p}, s_{g}, and s_{m} can be random variables [13], and fault paths that violate the conditions (212)–(215) in a given GKP ExRec are regarded as bad. Extending the techniques developed in this paper to such a generalized analysis is left to future work, but our analysis clarifies that taking nonzero s_{p}, s_{g}, and s_{m} satisfying these conditions suffices to show that $\tilde{C}$ experiences a local Markovian noise model.

By combining the threshold theorem of a qubit concatenated code with local Markovian noise in Ref. [38], we reach the following conclusion.

Corollary 37 (FT threshold under an (s,ϵ)- and (E,s,ϵ)-independent Markovian noise model)**.** Consider implementing an original circuit C on qubits using an FT-GKP circuit C' on CV systems. Suppose all preparations satisfy the E_{prep}-energy constraint and all gates satisfy the g_{sup}-energy constraint (Defs. 21 and 22). For $0<\epsilon<1$, consider a family of noise models on C' parameterized by $(s_{\mathrm{p}},s_{\mathrm{g}},s_{\mathrm{m}};\epsilon)$ in which $(s_{\mathrm{p}},\epsilon)$-independent Markovian noise is applied to GKP preparation gadgets, $(E^{\ell}_{\max}(\epsilon),s_{\mathrm{g}},\epsilon)$-independent Markovian noise is applied to GKP gate gadgets (including waits), and $(E^{\ell}_{\max}(\epsilon),s_{\mathrm{m}},\epsilon)$-independent Markovian noise is applied to GKP measurement gadgets (Defs. 8–10). Then, there exists a fault-tolerant threshold $\epsilon_{\mathrm{th}}>0$ such that for any $\epsilon<\epsilon_{\mathrm{th}}$ and any choice of the parameters $(s_{\mathrm{p}},s_{\mathrm{g}},s_{\mathrm{m}})$ satisfying Eqs. (212)–(215), we can achieve fault-tolerant quantum computation using a (noisy) CV circuit C' under an independent Markovian noise model parameterized by $(s_{\mathrm{p}},s_{\mathrm{g}},s_{\mathrm{m}};\epsilon)$. In particular, there exists a threshold $s_{\mathrm{th}}>0$ such that if $\epsilon<\epsilon_{\mathrm{th}}$ and $s_{\mathrm{p}},s_{\mathrm{g}},s_{\mathrm{m}}<s_{\mathrm{th}}$, then we can implement a fault-tolerant quantum computation that achieves a constant accuracy ε for a logical outcome of W-qubit D-depth circuit within $\mathcal{O}(\mathrm{poly}(\log_{\epsilon_{\mathrm{th}}/\epsilon}(WD/\varepsilon)))$ space and time overheads.

Proof. Since the qubit FT circuit has a fault-tolerant threshold $\epsilon^{\star}_{\mathrm{qubit}}$ against local Markovian noise [38], by applying it to $\tilde{C}$ in Theorem 36, the circuit C' can be made fault tolerant for any choice $(s_{\mathrm{p}},s_{\mathrm{g}},s_{\mathrm{m}};\epsilon)$ of parameters as long as the triple $(s_{\mathrm{p}},s_{\mathrm{g}},s_{\mathrm{m}})$ satisfies Eqs. (212)–(215) and $10\epsilon L_{\max}<\epsilon^{\star}_{\mathrm{qubit}}$, i.e., $\epsilon<\epsilon_{\mathrm{th}}$ with $\epsilon_{\mathrm{th}}\coloneqq\epsilon^{\star}_{\mathrm{qubit}}/(10L_{\max})$. The last statement directly follows by substituting $s_{\mathrm{p}}=s_{\mathrm{g}}=s_{\mathrm{m}}<c/38\eqqcolon\epsilon_{\mathrm{th}}$ to Eqs. (212)–(215), where c is in Eq. (6), and using the estimate of space and time overheads of qubit concatenated code against local Markovian noise model in Ref. [38]. Q.E.D.

Remark 38. One can bias threshold values of s for state preparations, gates, and measurements, as long as Eqs. (212)–(215) are satisfied. In particular, state preparations are expected to have more noise in typical experimental setup. We thus choose $13s_{\mathrm{p}}=s_{\mathrm{g}}=s_{\mathrm{m}}<c/182\eqqcolon\epsilon'_{\mathrm{th}}$, which results in $s_{\mathrm{p}}<\sqrt{\pi}/14$ and $s_{\mathrm{g}}=s_{\mathrm{m}}<\sqrt{\pi}/182$.

[1] M. H. Shaw, A. C. Doherty, and A. L. Grimsmo, Stabilizer Subsystem Decompositions for Single- and Multimode Gottesman-Kitaev-Preskill Codes, PRX Quantum **5**, 010331 (2024).
[2] A. S. Holevo, *Probabilistic and Statistical Aspects of Quantum Theory*, Vol. 1 (Edizioni della Normale Pisa, 2011).
[3] D. Gottesman, A. Kitaev, and J. Preskill, Encoding a qubit in an oscillator, Phys. Rev. A **64**, 012310 (2001).
[4] X. Zhou, D. W. Leung, and I. L. Chuang, Methodology for quantum logic gate construction, Phys. Rev. A **62**, 052316 (2000).
[5] J. Zak, Finite Translations in Solid-State Physics, Phys. Rev. Lett. **19**, 1385 (1967).
[6] J. Zak, Dynamics of Electrons in Solids in External Fields, Phys. Rev. **168**, 686 (1968).
[7] J. Zak, The kq-representation in the dynamics of electrons in solids, in *Solid State Physics*, Vol. 27 (Elsevier, 1972) pp. 1–62.
[8] G. Pantaleoni, B. Q. Baragiola, and N. C. Menicucci, Modular Bosonic Subsystem Codes, Phys. Rev. Lett. **125**, 040501 (2020).
[9] G. Pantaleoni, B. Q. Baragiola, and N. C. Menicucci, Subsystem analysis of continuous-variable resource states, Phys. Rev. A **104**, 012430 (2021).
[10] G. Pantaleoni, B. Q. Baragiola, and N. C. Menicucci, Hidden qubit cluster states, Phys. Rev. A **104**, 012431 (2021).
[11] G. Pantaleoni, B. Q. Baragiola, and N. C. Menicucci, Zak transform as a framework for quantum computation with the Gottesman-Kitaev-Preskill code, Phys. Rev. A **107**, 062611 (2023).
[12] S. Glancy and E. Knill, Error analysis for encoding a qubit in an oscillator, Phys. Rev. A **73**, 012325 (2006).
[13] D. Gottesman, An Introduction to Quantum Error Correction and Fault-Tolerant Quantum Computation (2009), arXiv:0904.2557 [quant-ph].
[14] H. Yamasaki and M. Koashi, Time-efficient constant-space-overhead fault-tolerant quantum computation, Nature Physics **20**, 247 (2024).
[15] T. Matsuura, H. Yamasaki, and M. Koashi, Equivalence of approximate Gottesman-Kitaev-Preskill codes, Phys. Rev. A **102**, 032408 (2020).
[16] E. Knill, Quantum computing with realistically noisy devices, Nature **434**, 39 (2005).
[17] A. Winter, Energy-constrained diamond norm with applications to the uniform continuity of continuous variable channel capacities (2017), arXiv:1712.10267 [quant-ph].
[18] M. E. Shirokov, Uniform continuity bounds for information characteristics of quantum channels depending on input dimension and on input energy, Journal of Physics A: Mathematical and Theoretical **52**, 014001 (2018).
[19] A. S. Holevo, Entanglement-assisted capacity of constrained channels, in *First International Symposium on Quantum Informatics*, Vol. 5128, edited by Y. I. Ozhigov, International Society for Optics and Photonics (SPIE, 2003) pp. 62 – 69.
[20] M. E. Shirokov and A. S. Holevo, On approximation of infinite-dimensional quantum channels, Problems of Information Transmission **44**, 73 (2008).

[21] A. Winter, Tight Uniform Continuity Bounds for Quantum Entropies: Conditional Entropy, Relative Entropy Distance and Energy Constraints, Communications in Mathematical Physics **347**, 291 (2016).
[22] S. Pirandola, R. Laurenza, C. Ottaviani, and L. Banchi, Fundamental limits of repeaterless quantum communications, Nature communications **8**, 1 (2017).
[23] M. E. Shirokov, On the Energy-Constrained Diamond Norm and Its Application in Quantum Information Theory, Problems of Information Transmission **54**, 20 (2018).
[24] M. Shirokov, Adaptation of the Alicki—Fannes—Winter method for the set of states with bounded energy and its use, Reports on Mathematical Physics **81**, 81 (2018).
[25] M. E. Shirokov and A. S. Holevo, Energy-Constrained Diamond Norms and Quantum Dynamical Semigroups, Lobachevskii Journal of Mathematics **40**, 1569 (2019).
[26] M. E. Shirokov, Advanced Alicki–Fannes–Winter method for energy-constrained quantum systems and its use, Quantum Information Processing **19**, 164 (2020).
[27] J. Watrous, *The Theory of Quantum Information*, 1st ed. (Cambridge University Press, USA, 2018).
[28] K. Sharma and M. M. Wilde, Characterizing the performance of continuous-variable gaussian quantum gates, Phys. Rev. Res. **2**, 013126 (2020).
[29] R. Nair, Quantum-Limited Loss Sensing: Multiparameter Estimation and Bures Distance between Loss Channels, Phys. Rev. Lett. **121**, 230801 (2018).
[30] R. Takagi, T. J. Yoder, and I. L. Chuang, Error rates and resource overheads of encoded three-qubit gates, Phys. Rev. A **96**, 042302 (2017).
[31] B. W. Walshe, B. Q. Baragiola, R. N. Alexander, and N. C. Menicucci, Continuous-variable gate teleportation and bosonic-code error correction, Phys. Rev. A **102**, 062411 (2020).
[32] C. Chamberland, P. Iyer, and D. Poulin, Fault-tolerant quantum computing in the Pauli or Clifford frame with slow error diagnostics, Quantum **2**, 43 (2018).
[33] N. C. Menicucci, Fault-Tolerant Measurement-Based Quantum Computing with Continuous-Variable Cluster States, Phys. Rev. Lett. **112**, 120504 (2014).
[34] B. Q. Baragiola, G. Pantaleoni, R. N. Alexander, A. Karanjai, and N. C. Menicucci, All-Gaussian Universality and Fault Tolerance with the Gottesman-Kitaev-Preskill Code, Phys. Rev. Lett. **123**, 200502 (2019).
[35] H. Yamasaki, T. Matsuura, and M. Koashi, Cost-reduced all-Gaussian universality with the Gottesman-Kitaev-Preskill code: Resource-theoretic approach to cost analysis, Phys. Rev. Res. **2**, 023270 (2020).
[36] S. G. Johnson, Saddle-point integration of c_∞ "bump" functions (2015), arXiv:1508.04376 [math.CV].
[37] D. J. A. McKechan, C. Robinson, and B. S. Sathyaprakash, A tapering window for time-domain templates and simulated signals in the detection of gravitational waves from coalescing compact binaries, Classical and Quantum Gravity **27**, 084020 (2010).
[38] P. Aliferis, An introduction to reliable quantum computation (2013), arXiv:1107.2148 [quant-ph].
